# The effects of estuary salinization on benthic assemblages: a richer and treacherous future

**DOI:** 10.1098/rsos.261145

**Published:** 2026-10-28

**Authors:** Francisco Barros, Yuri Costa, Marcos Krull, Wagner Magalhães, Sabrina Palma, Alice Reis, Gabriel Souza, Amanda Martins

**Affiliations:** ^1^ Laboratório de Ecologia Bentônica, IBIO, CIEnAM, INCT IN-TREE & PELD-BTS, Universidade Federal da Bahia, Salvador, Brazil; ^2^ Instituto de Biologia, Universidade Federal da Bahia, Salvador, Brazil; ^3^ Núcleo de Socioecologia Marinha, Universidade Federal da Bahia, Salvador, Brazil; ^4^ Centro de Ciências Agrárias, Universidade Federal do Recôncavo da Bahia, Cruz das Almas, Brazil; ^5^ Instituto Brasileiro do Meio Ambiente e dos Recursos Naturais Renováveis, Brasília, Brazil

**Keywords:** benthic macroinfauna, estuarine richness, tropical estuaries, long-term monitoring, salinity gradient, climate change

## Abstract

Estuarine salinization is emerging as a major consequence of climate change, yet its long-term ecological effects on tropical benthic assemblages remain poorly understood. Using nearly two decades of monitoring across three tropical estuaries, we evaluated temporal changes in benthic macroinfauna structure along their salinity gradients. We tested whether salinization would increase richness, change assemblage composition and cause shifts in benthic invertebrate distribution along the estuarine gradient. In two estuaries (Jaguaripe and Subaé) richness increased over time. Multivariate analyses showed that salinity remained the primary driver of benthic structure. Although some homogenization has occurred in middle and upper estuary zones in recent years, we found no consistent evidence that lower and middle estuarine taxa expanded landward. One estuary (Subaé) exhibited weaker temporal trends, possibly owing to historical contamination masking salinity effects. Our findings highlight that estuarine benthic assemblages are indicators of salinization, and increasing richness does not necessarily imply ecological improvement. Shifts in salinity regimes may compress estuarine zones, threaten oligohaline assemblages and alter ecosystem functions. Long-term monitoring, especially in tropical estuaries where climate extremes and human pressures are intensifying, is essential to detect tipping points and guide adaptive management and conservation strategies.

## 1. Introduction

Estuaries are natural connectors between terrestrial and oceanic systems, are among the most valuable ecosystems on Earth, and have supported human populations along their shores for thousands of years [1]. They were one of the principal sites of human settlement, owing to their ease of shipping access, availability of freshwater sources, fertile alluvial land for agriculture and abundance of fish and shellfish [2]. During the past century, the pace of urbanization and anthropogenic development has been greater in coastal watersheds and shoreline zones than in inland regions [3]. Furthermore, expanding world trade and the development of heavy industries have promoted the growth of ports and urban and industrial zones [4], with increasing land-use conversion, especially around estuaries.

Climate change is a major global issue, leading to sea level rise (SLR) and an increase in extreme climate events, such as droughts and floods, which are impacting estuaries. Climate impact drivers, such as warming, acidification, waves, storms, SLR and runoff, will have consequences for ecosystem function and services in estuaries [5], but with regional differences in the magnitude of change. Over the past decades, it has been well recognized that estuarine saline intrusion, resulting from sea-level rise, has been occurring [6,7]. Salinization may also cause the landward displacement and compression of tidal freshwater and oligohaline zones, particularly where their upstream migration is constrained by in-channel barriers such as dams, weirs or sluices. This process, termed estuarine squeeze, may ultimately lead to the loss of upper-estuarine transitional habitats [8]. In fact, the impacts of SLR, coupled with extreme drought events, will have profound ecological effects with serious consequences for future generations, especially in the Global South, owing to limited resources and adaptive capacity [9].

Biological assemblages are structured along estuarine gradients, with species distributions and abundances reflecting environmental tolerances [10–12], thus they can be affected by salinity regime changes [13]. Among the most ecologically relevant assemblages are benthic macrofauna, which are essential to estuarine ecosystem functioning [14]. By processing detritus, reworking sediments and recycling nutrients, benthic communities regulate biogeochemical cycles and sustain food webs [15–17]. Changes in salinity can also alter benthic functional traits, with associated changes in sediment biogeochemistry [18]. Their relative immobility and sensitivity to environmental stressors make them reliable bioindicators of ecological condition [19].

Even though changes in salinity can restructure benthic assemblages, and historical evidence shows that benthic macrofauna respond strongly to salinity fluctuations [20], the long-term effects of salinization on the structure and function of estuaries are still largely unknown [21]. Some studies have been done in temperate estuaries in Europe and North America [22–24], whereas tropical estuaries, where both biodiversity and human dependence on estuarine resources are particularly high, remain largely under-represented in the scientific literature. Therefore, while salinization is widely acknowledged as a critical driver of change in estuarine systems [25,26], its effects on the structure and functioning of benthic macrofauna in tropical estuaries remain poorly documented [27]. Moreover, few studies explicitly test whether benthic assemblages can serve as reliable indicators of salinization trends under ongoing climate change. Filling this gap is essential for both ecological understanding and applied management, as benthic responses could provide an early-warning system for detecting salinity-driven ecosystem shifts.

Estuaries are variable systems, with naturally high variability (i.e. salinity, sediments, suspended material, currents) and frequently associated with several anthropogenic activities (i.e. urbanization, pollution, dredging) [28]. Such spatial and temporal variability is intrinsic to a well-marked natural gradient and thus must be considered and treated as such. General comments on the adaptation of tropical coastal ecosystems to climate change (e.g. [29]) may foster discussion, but they barely provide data to actually understand contemporary changes in organisms, populations or communities. Seasonal variation in salinities in urbanized estuaries might be useful for creating predictions of the effects of salinization in estuarine benthic assemblages [21], but these are not factual long-term changes in these naturally complex systems. Furthermore, while manipulative experiments can aid in understanding species' capabilities to cope with saltier environments, long-lasting studies are mandatory. Ecological long-term monitoring of the structure and functioning of biological assemblages is essential for the timing of implementation of effective mitigation strategies. Nevertheless, it can take several decades for the monitoring-generated data to be useful. There are fewer funding opportunities, and it requires maintenance of resources, expertise and protocols. Thus, the initiation of long-term estuarine monitoring to assess the potential impacts of rising global temperatures, sea-level rise and resulting salinization is a ‘hot topic’, yet it is a ‘cold start’.

There is a traditional proposition of patterns of richness in estuaries with two peaks of richness at different points, one in the oligohaline zone (i.e. upper estuary) and another in the euhaline zone (lower estuary). However, this proposition was based on the Baltic Sea [20,30], which is quite a unique environment [31] and unlike most estuaries worldwide, salinity across this large, permanently brackish system is comparatively stable. A more consistent and general pattern is a decrease in richness of benthic assemblages from marine to freshwater [10,11,32,33], which was initially suggested for the tropical estuaries of Todos os Santos Bay (TSB). Accordingly, increasing salinity could allow taxa associated with saltier conditions to occur farther upstream and potentially increase estuarine richness. It has been reported that high salinity events (more than 35) have been occurring for decades in TSB [34], and a logical expectation is that the estuaries in this bay are getting saltier. In this paper, we used long-term monitoring data to evaluate whether benthic assemblages exhibit signs of estuarine salinization. Therefore, we assessed if salinization would: (i) facilitate the occupation of the middle and upper estuarine zones by lower estuarine species (H1), (ii) lead to an increase in the richness of estuarine benthic assemblages (H2), and (iii) change the gradient structure of the benthic assemblages over time (H3).

## 2. Material and methods

### 2.1. Sampling

In TSB, the second-largest bay in Brazil, tides are characteristically semi-diurnal and the currents are tide-driven [35]. TSB has rarely been mischaracterized as a single large estuary; in fact, salinity variation, from freshwater to marine water (i.e. estuarine system), is found only in the TSB tributaries (i.e. Jaguaripe, Paraguaçu and Subaé). TSB and its estuaries remain relatively well preserved [36]. Among them, the Jaguaripe estuary is considered the most pristine [37], whereas the Subaé estuary exhibits historical inorganic contamination in the upper estuary [38]. The Paraguaçu is a large hydrological system where freshwater input has been regulated by a dam since 1985 [39], and this regulatory control was intensified in 2005 with the commencement of an integrated hydroelectric power plant operation. Tidal influence in the Paraguaçu extends upstream to immediately below the Pedra do Cavalo dam, which is therefore located at or very close to the upstream tidal limit of the estuary. All sampling stations were located within the tidally influenced portion of the estuary. All three estuaries are bordered by extensive mangrove vegetation [12] and are used as a main source of income by many traditional communities [40].

The estuarine portion of the main tributaries of TSB was sampled in this study, including upper, middle and lower zones (i.e. Euhaline, Polyhaline, Mesohaline and Oligohaline zones, *sensu* Venice system). Macrozoobenthic assemblages from 10 (Jaguaripe and Paraguaçu) and 11 (Subaé) subtidal stations were sampled along the entire salinity gradient (figure 1). The stations were numbered nos. 1 to 10 or no. 11, where no. 1 was most seaward and no. 10 or no. 11 were the most landward. The Jaguaripe estuary was sampled six times (May 2006, August 2007, July 2010, August 2014, March 2019 and July 2022). The Paraguaçu estuary was sampled five times (May and December 2005, June 2011, August 2014 and October 2022), and the Subaé estuary was sampled six times (June 2004, March 2006, December 2009, April 2011, March 2013 and December 2022).

**Figure 1.**
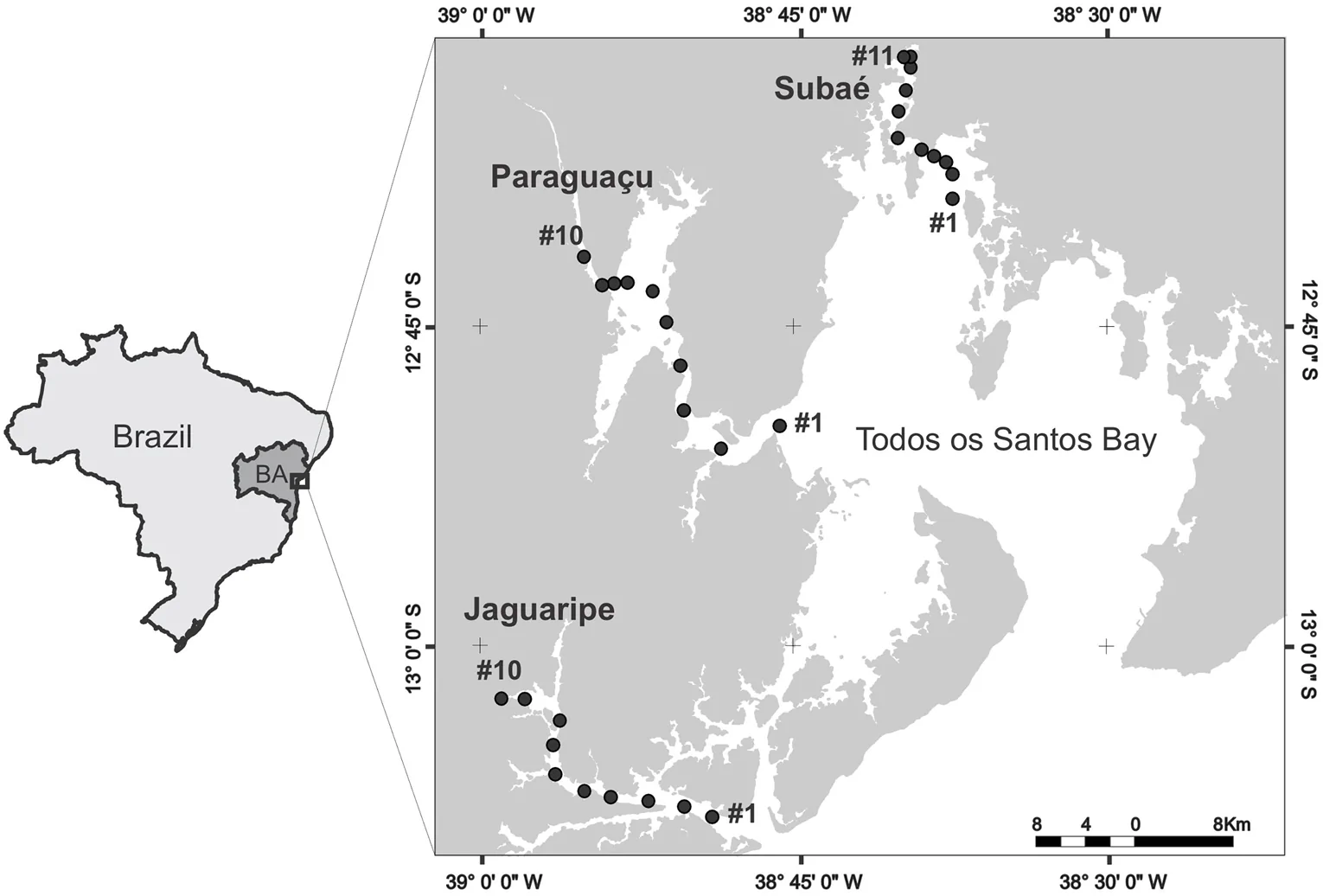
Sampling stations in the estuaries (Jaguaripe, Paraguaçu and Subaé) of Todos os Santos Bay, Bahia, Brazil.

In Jaguaripe and Subaé estuaries, four replicates were manually collected at two sites (around 20 m apart) at each station using a 0.008 m^2^ corer. In the Paraguaçu estuary, three replicates were collected at two sites (also around 20 m apart) at each station using a 0.05 m^2^ van Veen grab (see details in [11,38,39]). On one occasion in the Subaé estuary (December 2009), sampling was a combination of corers and grabs and different mesh sizes (see details in [41]).

All 1286 samples were sieved through a 0.5 mm mesh in the field and preserved in 70% alcohol until sorting in the laboratory (but see [41]; for Subaé 2009). Invertebrates were identified mostly to family, considering that family level is adequate to show estuarine macrofaunal changes [10,11,32,37,41–43].

One sediment sample was collected at each station, at each sampling occasion, for grain size analysis [44]. At each station, the salinity of the superficial water was measured using a Hydrolab DataSonde or a refractometer.

### 2.2. Data analysis

#### 2.2.1. General patterns

To visualize potential changes in richness and total abundances over time, the mean values were calculated for each station and for the three estuaries at each time. Patterns of the abundance of the most abundant taxa along the three estuaries on each sampling occasion were also inspected. To examine patterns of the temporal changes in assemblage composition, similarity percentages (SIMPER) analyses were evaluated for each station and each pair of consecutive surveys within each estuary. For each comparison, mean Bray–Curtis dissimilarity and the three taxa contributing most to temporal dissimilarity were recorded (supplementary material, table S3). The direction of change of each contributor was classified as increase, decline, appearance, disappearance or no change according to its change in abundance between surveys. To assess whether temporal changes were spatially consistent, station-level results were then summarized for each consecutive survey pair by identifying the taxa that recurred most frequently among the principal SIMPER contributors and counting the direction of their responses across stations (supplementary material, table S4). To visualize the general structure of the benthic assemblages along each estuarine gradient over time, abundances of all taxa were fourth root transformed and Bray–Curtis dissimilarities calculated to generate non-metric multidimensional scaling (nMDS).

#### 2.2.2. Structure of the benthic assemblages and environmental variables

To test for potential changes in the structure of the benthic assemblages along the estuarine gradient, we used a permutational multivariate analysis of variance (PERMANOVA). Previous studies in these estuaries showed that sites within a station were rarely different [11]; thus, these analyses were performed separately for each estuary with two factors: sampling occasion (fixed with six levels for Jaguaripe and Subaé and five levels for Paraguaçu) and stations (fixed with 10 levels for Jaguaripe and Paraguaçu and 11 levels for Subaé). For these analyses, benthic data were fourth root transformed (i.e. higher weight for composition instead of abundance), and Bray–Curtis dissimilarity matrices with a dummy variable (*n* = 1) were used. To test for significant differences in richness and for the changes in species abundances from the lower estuarine stations to the middle and upper estuarine stations at each estuary, a permutational ANOVA was used. These analyses were performed with Euclidean distance-based matrices with the same factors and levels as described above.

To evaluate the influence of salinity and grain size on the structure of the benthic assemblages over time, a distance-based redundancy analysis (dbRDA) was performed for each estuary. For this analysis, benthic assemblage replicates from each station were summed, data were fourth root transformed and Bray–Curtis dissimilarity matrices were used. Colinear variables (*r* > 0.8) were excluded, and environmental variables (grain size and salinities) were log(*x*+1) transformed. All analyses and figures were made using Primer 7. Figures were adjusted for clarity using standard graphic editing tools; only cosmetic changes were applied.

## 3. Results

### 3.1. General patterns

From a total of 152 different taxa, the most abundant were polychaetes from the families Nereididae (*n* = 3178; 15.6% of the total number of individuals), Magelonidae (1848; 9.1%), Cirratulidae (1617; 7.9%), Orbiniidae (976; 4.8%), Spionidae (873; 4.3%), Paraonidae (725, 3.6%) and Capitellidae (587; 2.9%) (supplementary material, figure S2). Bivalves from the family Tellinidae (2643; 13%) and isopods from the family Cirolanidae (553; 2.7%) were also abundant (supplementary material, figure S2). The SIMPER results showed that the taxa driving temporal dissimilarity varied markedly among stations, years and estuaries (supplementary material, tables S3 and S4), with recurrent taxa often showing contrasting directions of change. In Jaguaripe, Nereididae and other dominant contributors alternated among increases, declines, appearances and disappearances. Similarly, Tellinidae in Paraguaçu and Subaé showed contrasting responses among stations and periods. Thus, SIMPER did not indicate the occupation of the middle and upper estuarine zones by lower estuarine species taxa.

Salinity on all sampling occasions in all estuaries decreased from the lower to the upper estuary, and generally higher values were observed in more recent sampling occasions (supplementary material, figure S3). We observed a greater proportion of coarse sands in the upper estuarine zones of the three estuaries (supplementary material, figure S4). The Jaguaripe estuary had a higher proportion of fine sands compared to Paraguaçu and Subaé estuaries, which in turn contained more mud (less than 0.062 mm). Over time, sediment grain sizes at each station along the estuarine gradients were relatively stable, with greater variability in the Subaé estuary (supplementary material, figure S4).

Benthic macroinfauna richness generally decreased from the lower to the upper estuary, but with large variability (figure 2). Richness in the Jaguaripe estuary showed a significant (permutational ANOVA, *p* < 0.05) increase from the first sampling occasions (2006, 2007) to the latter sampling occasions (2019, 2022). Richness in Paraguaçu estuary also significantly increased from the two samplings conducted in 2005 to the latter ones (2011, 2014 and 2022). The Subaé estuary showed a relatively more complex pattern; richness was lower in the earlier sampling dates (2004, 2006) than on the more recent one (2022), but in 2009, richness was also significantly higher than in the other sampling times. No consistent patterns of occupation of the middle and upper estuarine zones by lower estuarine species were observed (supplementary material, figure S2a–g).

**Figure 2.**
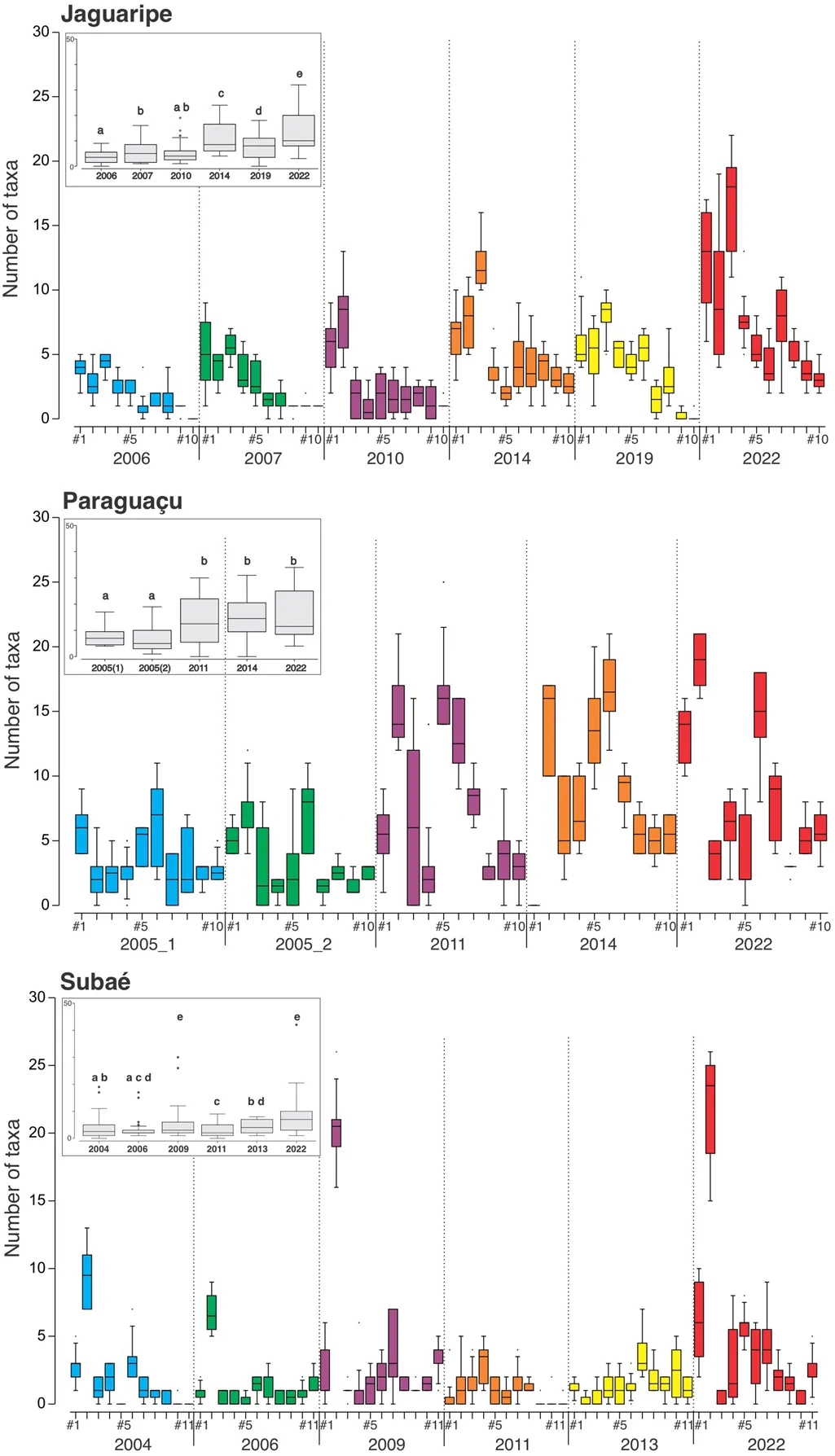
Richness (number of taxa) of the benthic assemblages on all stations of each estuary at each sampling occasion (different colours). Top graph: Jaguaripe estuary, middle graph: Paraguaçu estuary, and lower graph: Subaé estuary. The inset shows the values of richness for the entire estuary on each sampling occasion (i.e. years), and the letters indicate the different groups (i.e. different letters indicate significant differences).

### 3.2. The structure of the benthic assemblages and environmental variables

Despite the maintenance of some similarity among sampling stations at different estuarine zones (supplementary material, figure S1), the structure changed over time in the estuaries of Jaguaripe and Paraguaçu, while no clear trend was observed in Subaé estuary (figure 3). The PERMANOVA showed significant differences among sampling occasions, stations and the interaction between them, in all estuaries (supplementary material, table S1). The pairwise test showed that, in the first sampling date in Jaguaripe estuary, benthic assemblages were different among stations in the middle part of the estuarine gradient (nos. 6–7, nos. 6–8; supplementary material, table S1; figure 3, top graph). In the following sampling occasion, similarities were also observed in the upper estuary stations. This pattern (middle and upper stations) was also observed in the subsequent years, but in 2022, the upper estuary (i.e. the euhaline zone) (no. 1, no. 2) also showed non-significant differences (supplementary material, table S1).

**Figure 3.**
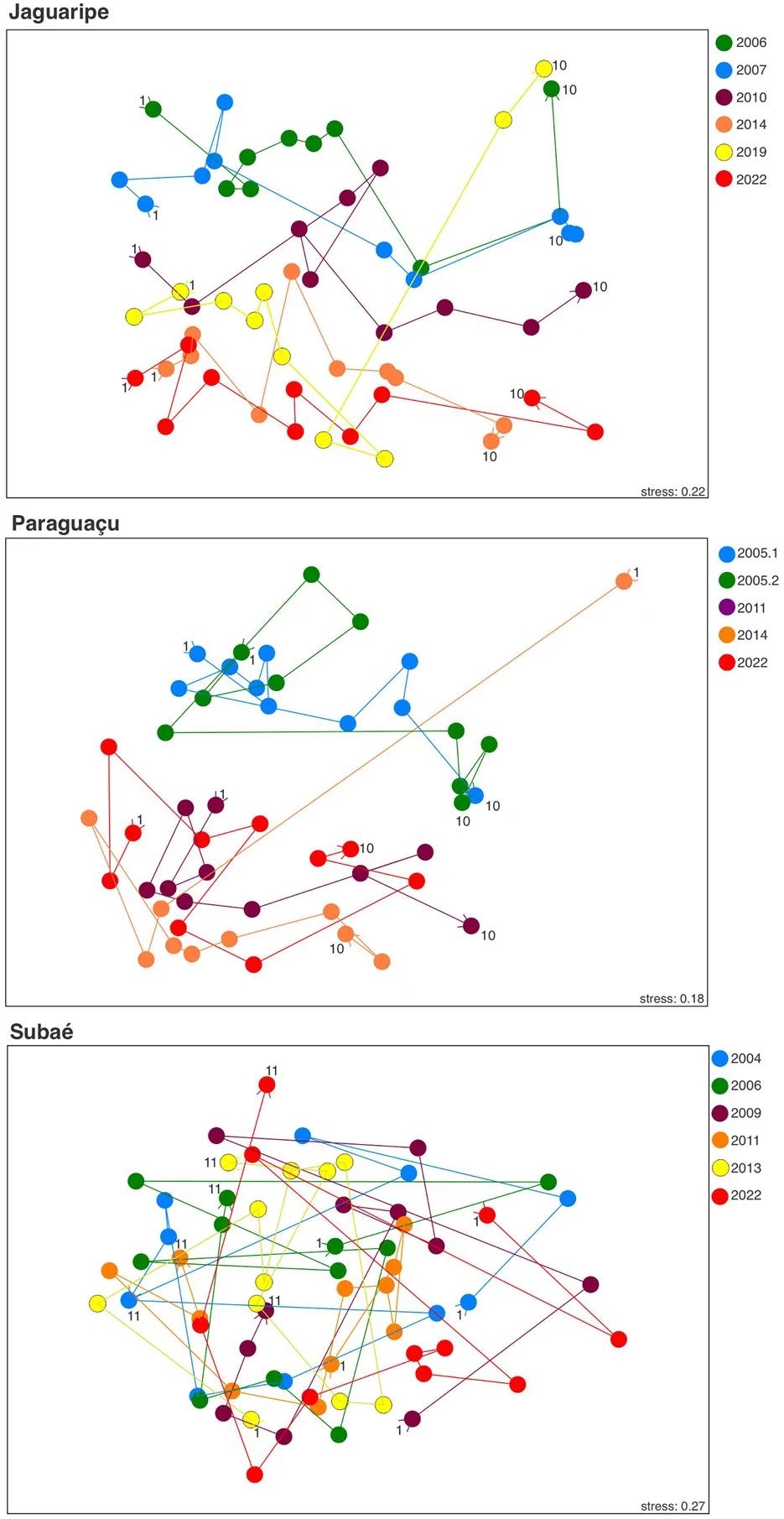
nMDS ordinations of benthic assemblages in each estuary. Top graph: Jaguaripe estuary, middle graph: Paraguaçu estuary, and lower graph: Subaé estuary. Each panel represents one estuary and colours indicate sampling occasions. Within each sampling occasion, stations are connected sequentially from station 1 (lower estuary) to station 10 or 11 (upper estuary). Consequently, the connected points of each colour represent the spatial assemblage gradient along the estuary, whereas differences in their position among colours represent temporal changes in assemblage structure.

Benthic assemblages sampled on the first three sampling occasions in the Paraguaçu estuary showed some similarities between stations located along the estuarine gradient (i.e. lower, middle and upper estuarine stations; figure 3; supplementary material, table S1). In 2014, the only non-significant result was between station nos. 8 and 9, whereas in 2022, similarities were restricted to the lower and middle portions. The Subaé estuary showed a relatively more homogeneous benthic assemblage over the years, especially in 2011 and 2013 (figure 3). However, in 2022, most of the stations significantly differed, except for two comparisons (no. 3 versus no. 10, no. 6 versus no. 7; supplementary material, table S1).

The dbRDA model for Jaguaripe estuary (figure 4, top), fitted with six abiotic variables (salinity, very coarse sand, coarse sand, medium sand, very fine sand and fine sediments < 0.063 mm), explained 34.7% of the total variation (supplementary material, table S2). The first two dbRDA axes accounted for 28% of the total variation (58% and 22% of the fitted variation, respectively). The first axis in dbRDA reflects a strong gradient of salinity (positive) opposed to coarse sand (negative correlation), whereas the second axis represents a contrast between fine sediments (negative) and very fine sand (positive). Thus, community structure is primarily structured by salinity and coarse sand, with contributions from other sediment grain sizes. For the Paraguaçu estuary (figure 4, middle), the model was fitted with eight variables (i.e. salinity, granule, very coarse sand, coarse sand, medium sand, fine sand, very fine sand and fine sediments), which explained 37% of the total variation (supplementary material, table S2). The first two dbRDA axes accounted for 27.5% of the total variation (40.8% and 33.4% of the fitted variation). The dbRDA1 reflects a strong gradient of fine sediments and salinity (positive), whereas dbRDA2 represents a contrast between granule (negative) and very coarse sand (positive). In Subaé (figure 4, bottom), the model with seven variables (salinity, pebble, very coarse sand, coarse sand, fine sand, very fine sand and fine sediments) explained relatively less of the total variation (24.9%) (supplementary material, table S2). The two dbRDA explained 10% and 6.1%, respectively, and the most important variables were salinity and coarse sand (supplementary material, table S2).

**Figure 4.**
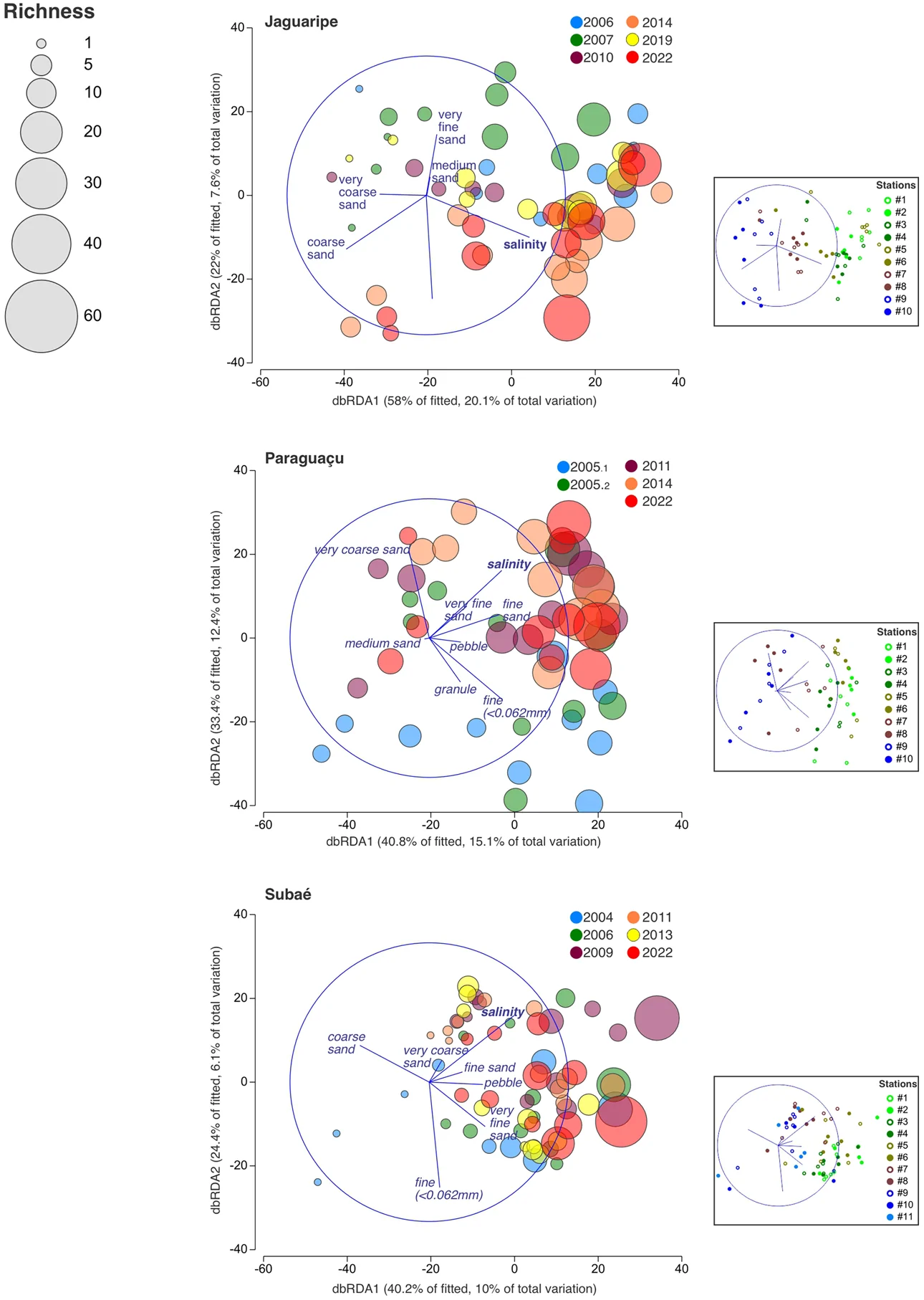
dbRDA of the estuaries: Jaguaripe (top), Paraguaçu (middle) Subaé (bottom). Richness of the benthic macroinfauna at each station is represented by the size of the bubbles according to the scale in the upper left. Sampling occasion is represented by different colours. On the right side, the inset dbRDA shows the sampling stations, where stations from the lower estuary are in bright green and in the upper estuary are in blue.

## 4. Discussion

Salinity was associated with the structure of the benthic assemblages at each sampling time, that is, differences between the upper, middle and lower estuary were clear, especially in two out of three systems. We did not observe a consistent upstream expansion of lower-estuarine taxa (H1), that is, important taxa (i.e. contributors to dissimilarities) frequently showed contrasting directions of change among stations, and responses often reversed between consecutive periods. However, there were marked changes in the structure of the benthic assemblages among sampling occasions (H3) and a clear increase in richness over the years (H2), although these trends were more pronounced in Jaguaripe and Paraguaçu. The former, is a less impacted estuarine system, whereas the latter has experienced stronger salinity increases, potentially intensified by reduced freshwater input owing to a dam that serves both water supply and hydroelectric generation [39]. Increasing salinization in estuaries worldwide is probably driving shifts in benthic assemblage structure [21,45,46].

In estuaries exposed to multiple stressors, assemblage response to salinization might be masked by other human impacts (e.g. pollution and dredging). Understanding the combined effects of these overlapping drivers on estuarine biodiversity will require well-designed studies. For instance, the Subaé estuary is the smallest studied estuary of TSB and has a history of pollution in its upper region [36], and this could have influenced the observed patterns. Manipulative experiments simulating future scenarios are needed, but monitoring is essential to assess whether experimental finding's hold under field conditions, detect cascade effects, phase shifts or tipping points and inform mitigation practices.

Benthic assemblages are useful indicators of ecological responses to estuarine salinization. The structure of these assemblages changed over the salinity gradient and was a good descriptor of change over the years. Salinization can be associated with changes in habitat structure (i.e. increase in complexity) which can also increase diversity [47]. Yet, salinization has been observed in some European estuaries [22,23] and increases in richness and in the extension of marine species in the upper part have been reported [47,48]. Our results differ in that increased richness was not accompanied by a consistent upstream expansion of lower-estuarine taxa. In tropical areas in the Global South, where higher temperatures and intense evapotranspiration combine with extreme droughts, salinization effects could be even more pronounced; however, relatively little research has been done on these systems.

Salinity was a major driver of the structure of the benthic assemblages in the studied estuaries, despite substantial temporal changes and the variability within estuarine systems. If middle estuarine zones move landwards, this could result in the salinization of neighbouring freshwater systems. However, if estuaries are modified, for example by freshwater inputs controlled by dams, as in Paraguaçu [39], or by other interventions, such as urban development, mesohaline and oligohaline regions may become intermittent or disappear. This process is consistent with the concept of *estuarine squeeze*, in which saline intrusion displaces tidal freshwater and oligohaline zones landwards while barriers constrain their upstream migration, potentially resulting in the compression or loss of these transitional zones [8]. The issue here is that these zones are characteristically inhabited by specialized and frequently dominant taxa. Although saline zones may be shifting within the studied estuaries, the underlying sediment characteristics remained largely unchanged over time. This mismatch is likely to affect the availability of substrate types required by some benthic species. However, the absence of a consistent landward expansion of lower-estuarine taxa in our data indicates that compression of salinity zones does not necessarily result in a simple upstream displacement of entire benthic assemblages, although long-term datasets with species-level identification might be more appropriate for examining such expansion. Additionally, experimental work and monitoring must address whether benthic functions will be taken over by a diverse marine assemblage and how this change will affect estuarine ecosystems [14,16,49,50]. Future studies should explore how salinization combined with other stressors, such as dam operations and pollution, may affect the structure and functions of the upper-estuarine benthic assemblages and experimentally test potential changes in trait combinations [51].

The observed changes will have effects on estuarine functions [14,16,50], but precise predictions are needed. Changes in salinity can alter benthic functional traits and associated sediment biogeochemical processes [18], reinforcing the potential functional consequences of salinization. We need to determine which groups of species are responsible for each estuarine function and how changes in salinity will affect not only the species, but also physicochemical processes and the interactions between them. For instance, estuaries with maximum turbidity zones will be affected, and changes in suspended particulate matter, flocculation and resuspension, which play an important role in estuarine and coastal ecology [52], will affect fauna and flora.

One of the most important natural connections between land and sea, that is, estuaries, is being affected by climate change. Changes in salinity regimes are considered a major hydrological modification in estuarine systems [53]. In the next decades, especially in regions where rainfall will decrease [54] concomitant with a SLR, we will probably see an estuarine gradient shrink, particularly in places where geomorphology or urban development will impede an estuarine zone's displacement to higher up freshwater systems [8]. The consequences for ecosystem functions and services must be modelled, and mitigation actions and restoration must be planned, with wide stakeholder involvement. This must include traditional communities whose subsistence practices (e.g. fishing and shellfish gathering) and long-term traditional ecological knowledge [40,55] are closely tied to estuarine environments and who are likely to be affected earlier than other social groups.

## Supplementary Material



## Data Availability

Occurrence data and metadata are public at [56]. Supplementary material is available online [57].
