# Supplementary material for: The effects of estuary salinization on benthic assemblages: a richer and treacherous future
Source: R Soc Open Sci. 2026 Oct 28;13(10):261145. doi: 10.1098/rsos.261145 (PMC13714414; doi:10.1098/rsos.261145)
Supplement: Supplementary file 1 [file rsos.261145.zip › rsos.261145/rsos261145_si_001.pdf]

SUPLEMENTARY MATERIAL

The effects of estuary salinization on benthic assemblages: a richer and treacherous future.

Francisco Barros, Yuri Costa, Marcos Krull, Wagner Magalhães, Sabrina Palma, Alice Reis, Gabriel Souza, Amanda Martins

Royal Society Open Science

10.1098/rsos.261145

Figure 1S. Boostrapped nMDSs of the three estuaries (Jaguaripe, Paraguaçu and Subaé) showing that in spite of the changes in the structure of the different estuarine zones. from the lower (#1) to the uper estuary #10). are dissimilar from each other some of the structure of the benthic assemblages was maintained along the different sampling occasions. In these analyses, benthic assemblages data from replicates of sites were summed and sites (n= 2) at each station at each sampling occasion were bootstrapped to generate the dispersions of the points. Centroids (av) of each estation (#1 to #10 or #11) are showed in black symbols

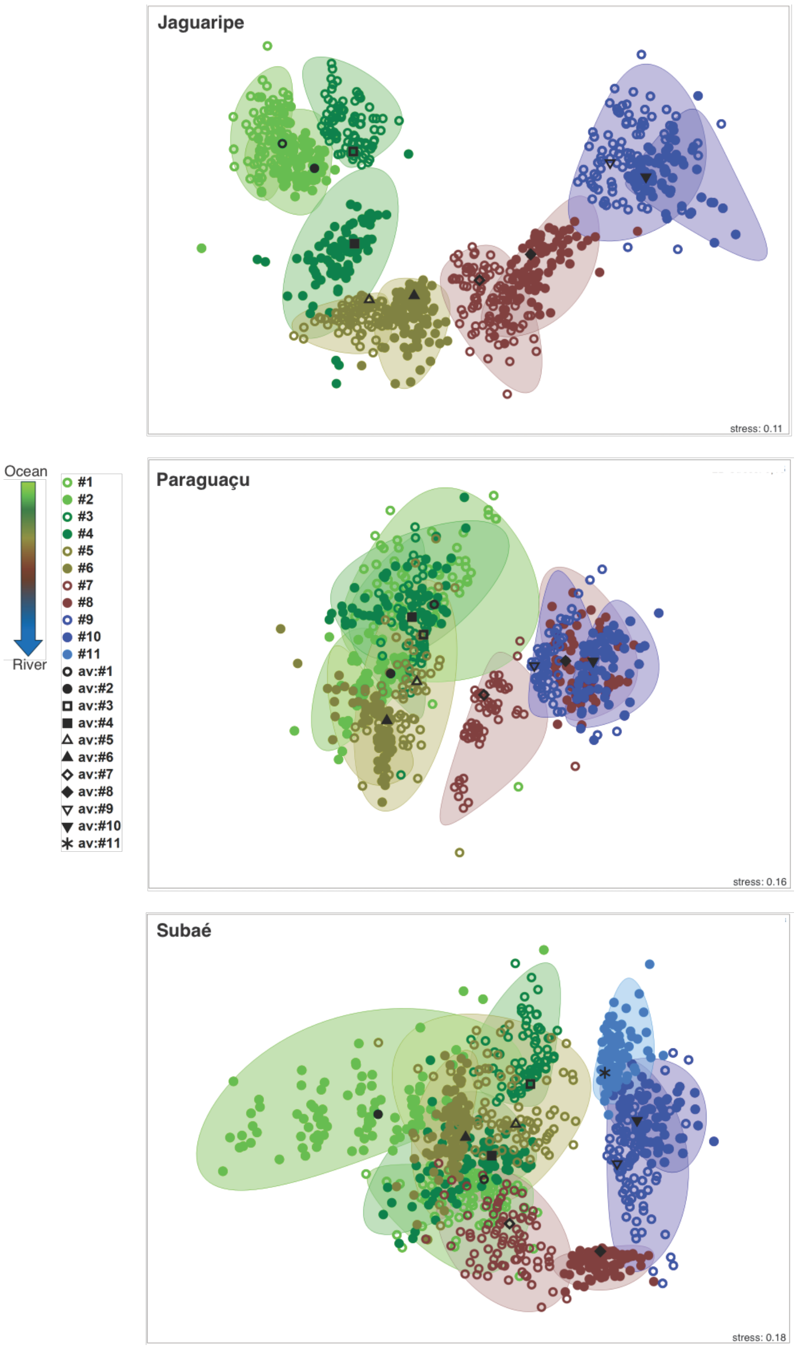

Figures 2A to G. Mean number of individuals of the seven most abundant taxa on each station of each estuary at the different sampling occasions.

A

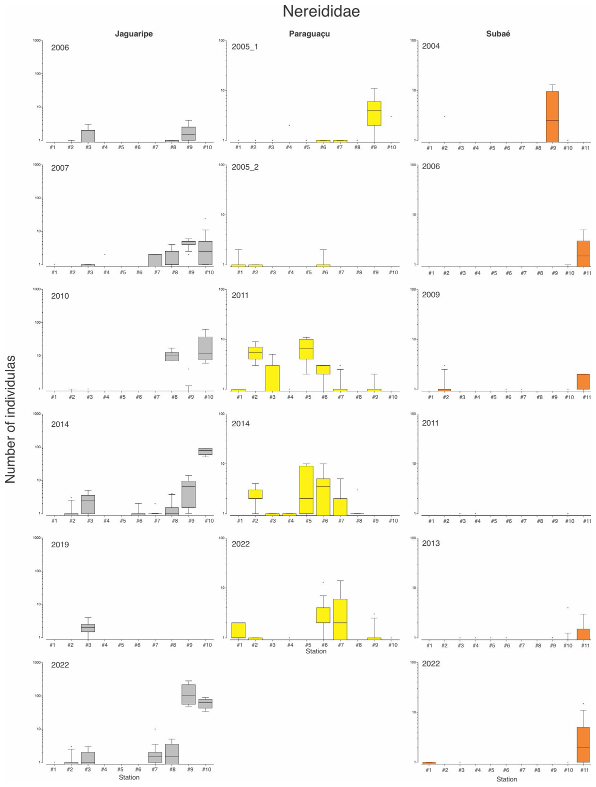

B

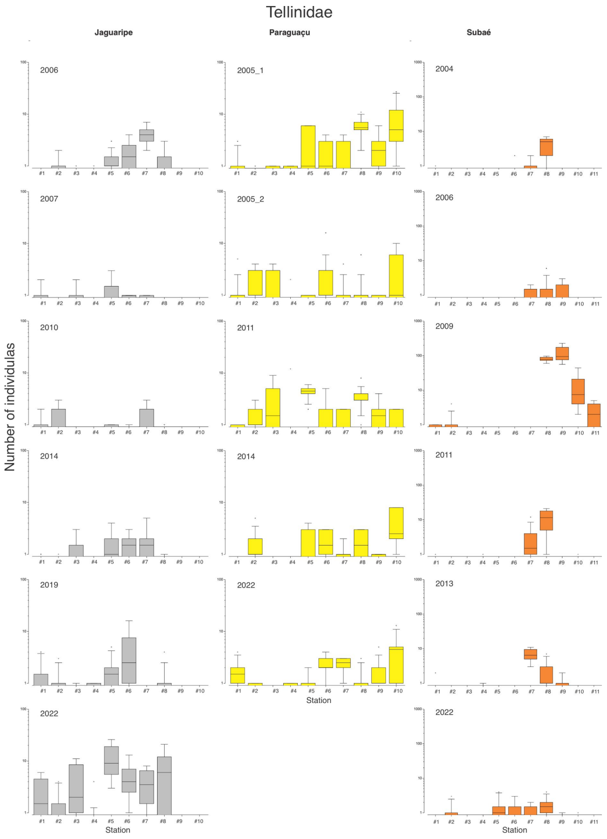

C

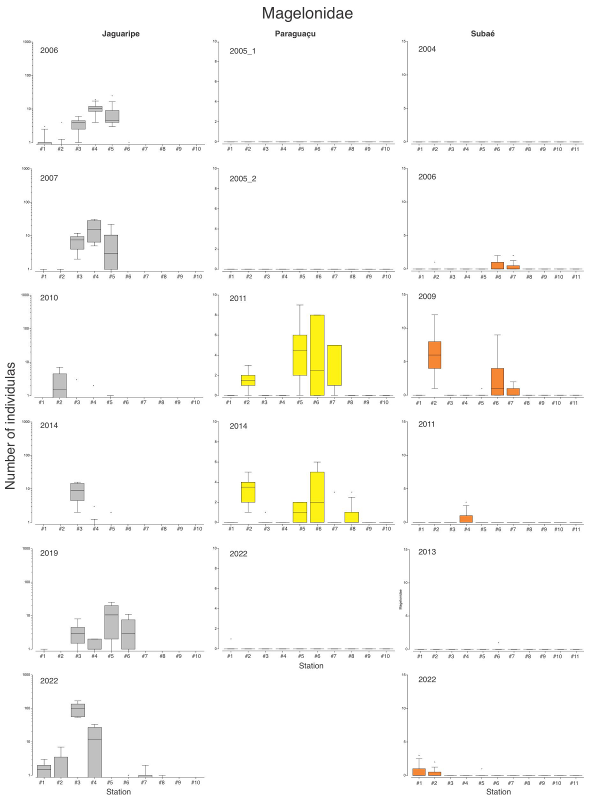

Magelonidae
Jaguaripe
Paraguaçu
Subaé
2006
2007
2010
2014
2019
2022
2005_1
2005_2
2011
2014
2022
2004
2006
2009
2011
2013
2022
Number of individulas
Station

D

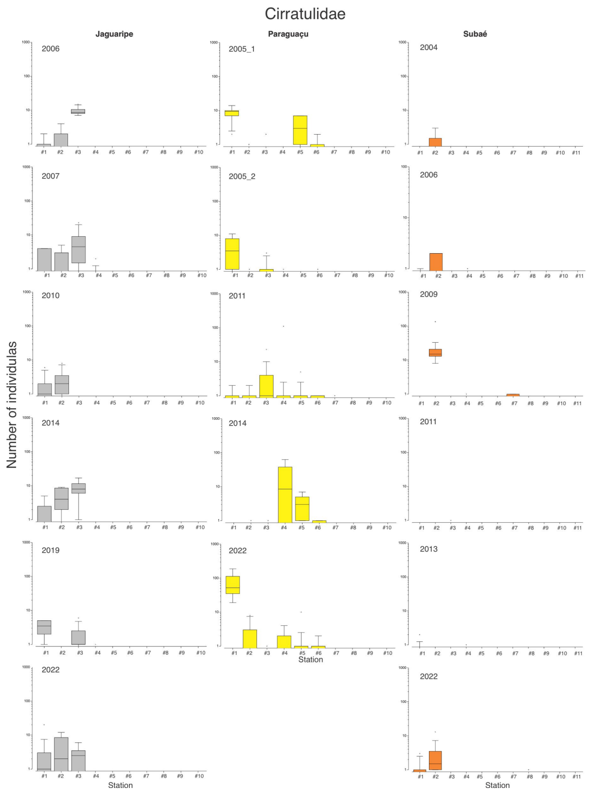

Cirratulidae
Jaguaripe
Paraguaçu
Subaé
2006
2007
2010
2014
2019
2022
2005_1
2005_2
2011
2014
2022
2004
2006
2009
2011
2013
2022
Number of individulas
Station

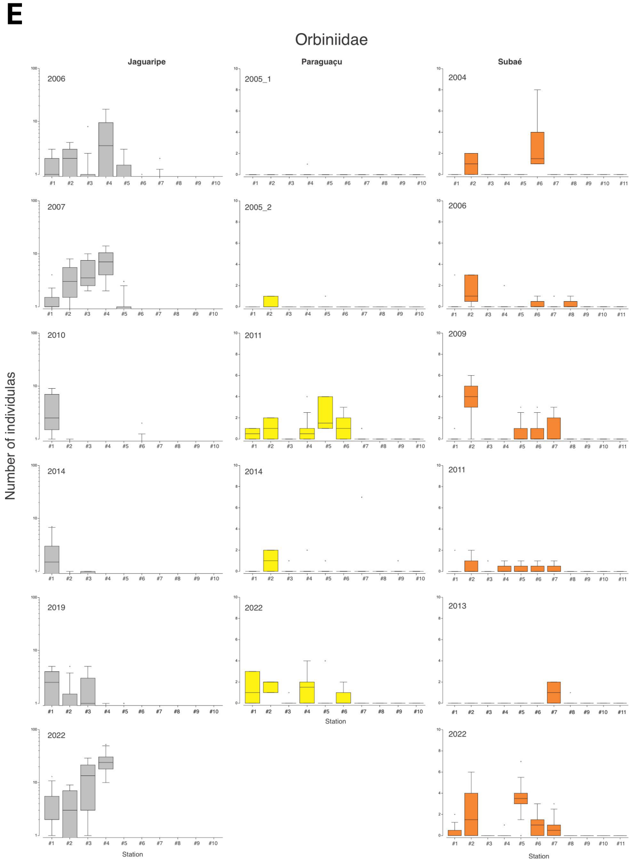
E
Orbiniidae
Jaguaripe
Paraguaçu
Subaé
Number of individulas
Station

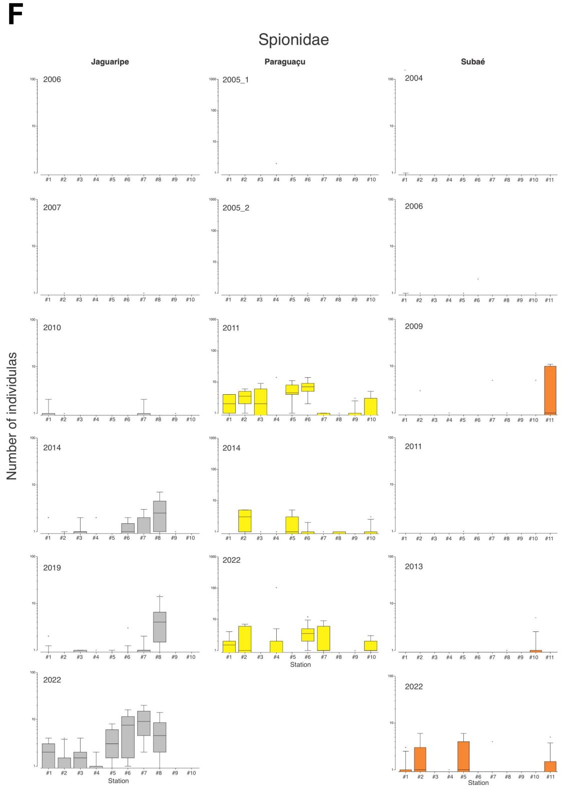
F
Spionidae
Jaguaripe
Paraguaçu
Subaé
Number of individulas
Station

G

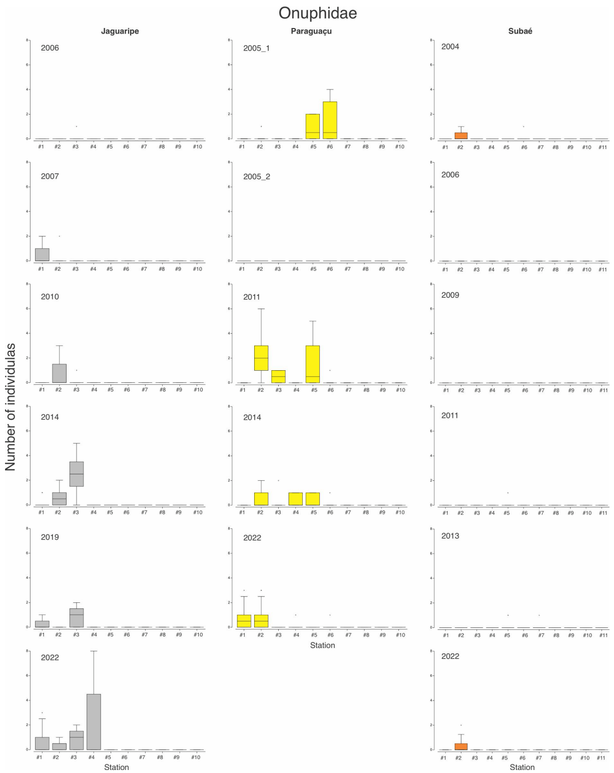

Onuphidae
Jaguaripe
Paraguaçu
Subaé
2006
2007
2010
2014
2019
2022
2005_1
2005_2
2011
2014
2022
2004
2006
2009
2011
2013
2022
Number of individulas
Station
Station
Station

Figure 3S. Water salinity observed at each station on each sampling occasion in the three estuaries. Box plots on the right show the values of salinity for each year.

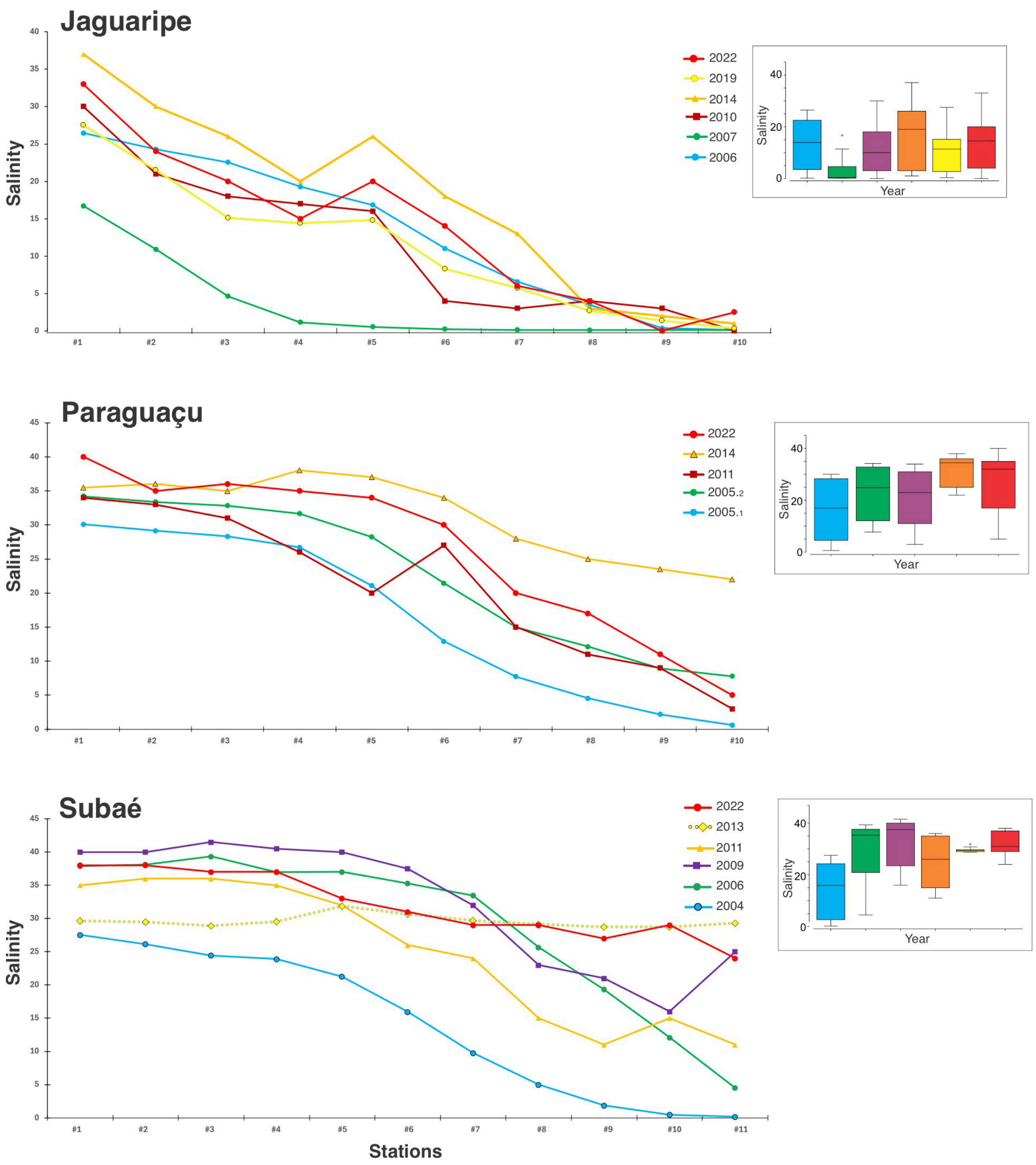

Figure 4S. Contribution of each grain size class at each station on each sampling occasion at each estuary.

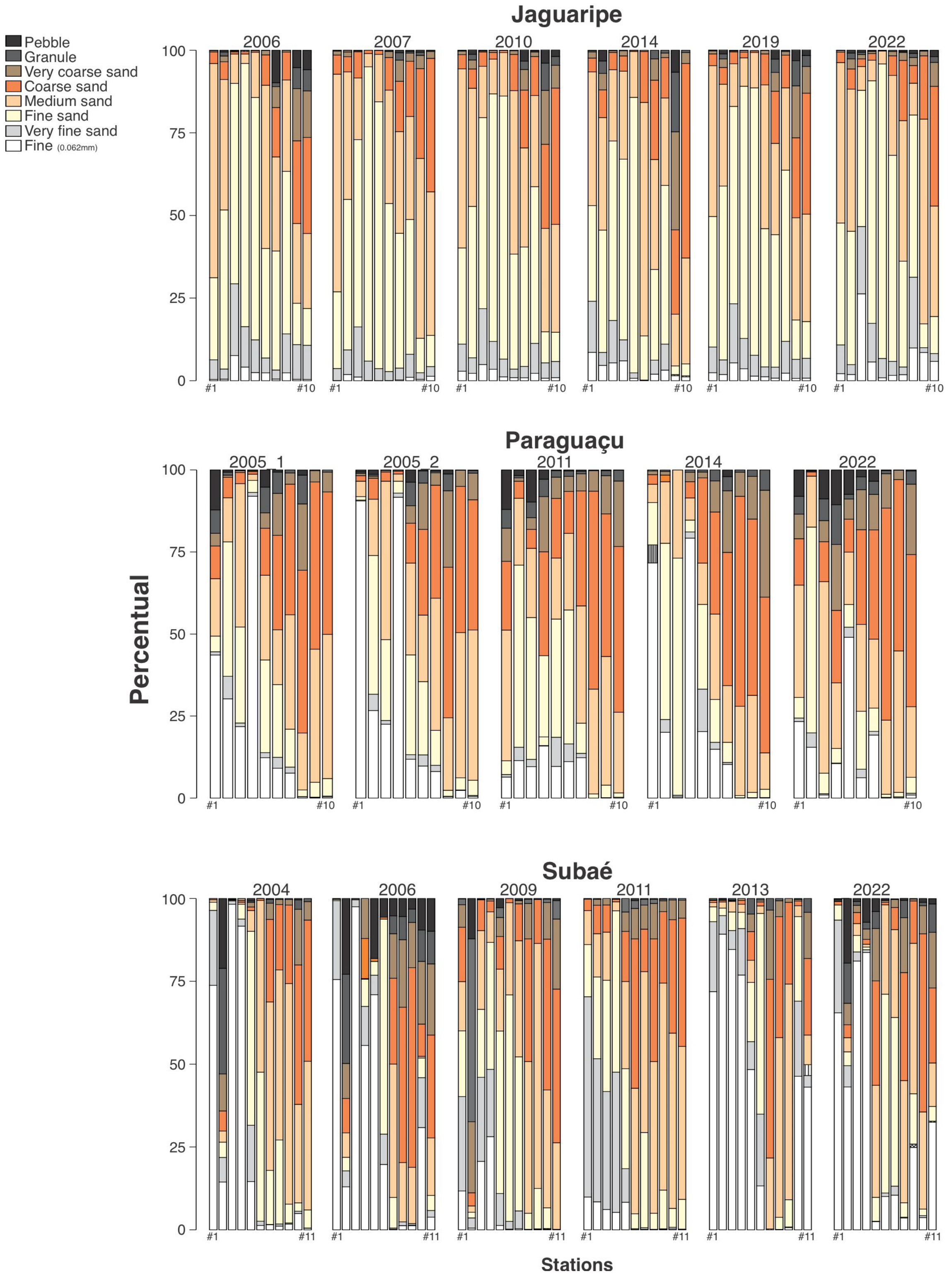

Table 1S. PERMANOVA and pairwise test results for each estuary. The non significant pairwise comparisons between stations within each year are show in parentheses.

a) Jaguaripe

Source	df	MS	Pseudo-F	P(perm)	R^2
**Year (Ye)**	5	34,634.0	39.597	0.0001	0.151
**Station (St)**	9	37,302.0	42.647	0.0001	0.292
**Ye x St**	45	6,078.3	6.9492	0.0001	0.238
**Res**	420	874.7			0.320
**Total**	479				1.000

**Non-significant pairwise**	
**2006**	(#6, #7) (#6, #8)
**2007**	(#6, #7) (#8, #10) (#9, #10)
**2010**	(#3, #4, #5, #6) (#3, #9) (#5, #6, #7)
**2014**	(#6, #7, #8)
**2019**	(#7, #8) (#9, #10)
**2022**	(#1, #2) (#5, #6) (#9, #10)

b) Paraguaçu

Source	df	MS	Pseudo-F	P(perm)	R^2
**Year (Ye)**	4	25,302.0	18.226	0.001	0.124
**Station (St)**	9	17,950.0	12.93	0.001	0.199
**Ye x St**	36	5,655.6	4.0739	0.001	0.250
**Res**	250	1388.3			0.427
**Total**	299				1.000

**Non-significant pairwise**	
**2005(1)**	(#2, #3, #4) (#2, #4, #6) (#2, #3, #7) (#7, #8) (#7, #9)
**2005(2)**	(#1, #3) (#2, #3) (#3, #4, #5, #7, #9) (#8, #9) (#8, #10)
**2011**	(#1, #3) (#1, #3, #4) (#1, #9) (#2, #3) (#2, #5, #6) (#3, #8, #9) (#3, #4, #9) (#9, #10)
**2014**	(#8, #10)
**2022**	(#3, #4, #5) (#4, #7) (#5, #8, #9) (#6, #7)

c) Subaé

Source	df	MS	Pseudo-F	P(perm)	R^2
**Year (Ye)**	5	13,263.00	14.926	0.001	0.073
**Station (St)**	10	17,165.00	19.317	0.001	0.190
**Ye x St**	50	5,528.90	6.2223	0.001	0.305
**Res**	440	888.56			0.432
**Total**	505				1.000

Non-significant pairwise	
**2004**	(#3, #4) (#4, #7) (#5, #10, #11) (#7, #8)
**2006**	(#1, #3, #4, #5, #8, #9) (#1, #4, #6) (#3, #7, #8, #9)
**2009**	(#1, #3, #4, #5) (#1, #6) (#5, #6, #7) (#8, #9)
**2011**	(#1, #2, #3, #5, #6) (#1, #3, #5, #6, #9, #10, #11) (#2, #11)
**2013**	(#1, #2, #3, #4, #5, #6) (#1, #9, #11) (#1, #3, #4, #5, #6, #11) (#4, #9, #10, #11) (#5, #9, #11) (#8, #9)
**2022**	(#3, #10) (#6, #7)

Table 2S. Results of the dbRDA analysis for each estuary.

a) Jaguaripe

Specified solution	R2	RSS	N. Vars
	0.34712	101730.00	6 3-5;7-9

Marginal tests						% of var. expl. by ind. axes			
						% out of fitted model		% out of total variation	
Variable	SS (trace)	Pseudo-F	P	Prop.	Axis	Ind.	Cum.	Ind.	Cum.
Very Coarse Sand	17750	7.4562	0.001	0.11391	1	57.95	57.95	20.12	20.12
Coarse Sand	24381	10.758	0.001	0.15646	2	21.97	79.92	7.63	27.74
Medium Sand	5077.9	1.9537	0.049	0.032587	3	9.97	89.9	3.46	31.2
Very Fine Sand	5495.8	2.1204	0.029	0.03527	4	4.29	94.19	1.49	32.69
Fine	8426	3.3156	0.002	0.054074	5	3.44	97.63	1.2	33.89
Salinity	28029	12.721	0.001	0.17988	6	2.37	100	0.82	34.71

Sequential tests							
Variable	R^2	SS (trace)	Pseudo-F	P	Prop.	Cumul.	res.df
Very Coarse Sand	0.11391	17750	7.4562	0.001	0.11391	0.11391	58
Coarse Sand	0.17212	9070	4.0076	0.001	0.058207	0.17212	57
Medium Sand	0.21966	7407.6	3.4115	0.001	0.047538	0.21966	56
Very Fine Sand	0.24103	3330.4	1.5488	0.123	0.021373	0.24103	55
Fine	0.30893	10581	5.306	0.001	0.067903	0.30893	54
Salinity	0.34712	5950.9	3.1003	0.003	0.03819	0.34712	53

Relationships between dbRDA coordinate axes and orthonormal X variables (multiple partial correlations)						
Variable	dbRDA1	dbRDA2	dbRDA3	dbRDA4	dbRDA5	dbRDA6
Very Coarse Sand	**-0.334**	0.008	0.526	-0.363	0.509	0.47
Coarse Sand	-0.575	-0.386	0.218	-0.174	-0.116	-0.655
Medium Sand	0.024	0.132	0.654	0.738	-0.078	-0.064
Very Fine Sand	**0.076**	0.438	0.352	-0.461	-0.682	0.035
Fine	0.043	-0.742	0.077	0.059	-0.448	0.487
Salinity	0.741	-0.301	0.345	-0.278	0.239	-0.326

	Weights (Coefficients for linear combinations of X's in the formation of dbRDA coordinates)					
Variable	dbRDA1	dbRDA2	dbRDA3	dbRDA4	dbRDA5	dbRDA6
Very Coarse Sand	-2.0336	4.6335	8.9391	-1.9845	5.3542	6.9362
Coarse Sand	-12.278	-12.278	-2.8582	-6.8262	-2.9427	-10.348
Medium Sand	6.1009	9.1386	12.477	10.557	-1.6596	4.0391
Very Fine Sand	0.034651	15.389	7.9879	-4.934	-6.1238	-1.6316
Fine	-1.3302	-18.389	0.30229	4.0658	-2.8634	5.6982
Salinity	11.354	-5.6591	2.8479	-4.7422	2.035	-4.5469

b) Paraguaçu

Specified solution		
R2	RSS	N. Vars
0.37047	79406.00	8 1-3;5-9

Marginal tests

Variable	SS (trace)	Pseudo-F	P	Prop.
Pebble	5938.4	2.3715	0.013	0.04708
Granule	2802.3	1.0906	0.341	0.022216
Very Coarse Sand	6616.6	2.6573	0.007	0.052456
Medium Sand	3396.8	1.3284	0.201	0.02693
Fine Sand	11275	4.7116	0.001	0.089385
Very Fine Sand	11410	4.7738	0.001	0.090458
Fine Sand	13286	5.6509	0.001	0.10533
Salinity	15121	6.5377	0.001	0.11988

% of var. expl. by ind. axes

	% out of fitted model		% out of total variation	
Axis	Ind.	Cum.	Ind.	Cum.
1	40.79	40.79	15.11	15.11
2	33.38	74.17	12.37	27.48
3	9.52	83.68	3.53	31
4	6.2	89.88	2.3	33.3
5	5.03	94.92	1.86	35.16
6	2.4	97.32	0.89	36.05
7	1.79	99.1	0.66	36.71
8	0.9	100	0.33	37.05

Sequential tests

Variable	R^2	SS (trace)	Pseudo-F	P	Prop.	Cumul.	res.df
Pebble	0.04708	5938.4	2.3715	0.013	0.04708	0.04708	48
Granule	0.069348	2808.9	1.1246	0.321	0.022269	0.069348	47
Very Coarse Sand	0.17136	12867	5.6629	0.001	0.10201	0.17136	46
Medium Sand	0.18198	1340.1	0.58444	0.866	0.010624	0.18198	45
Fine Sand	0.26395	10339	4.8998	0.001	0.081967	0.26395	44
Very Fine Sand	0.27662	1598	0.75308	0.739	0.012669	0.27662	43
Fine Sand	0.29882	2800.9	1.3301	0.165	0.022206	0.29882	42
Salinity	0.37047	9037	4.6661	0.001	0.071645	0.37047	41

Relationships between dbRDA coordinate axes and orthonormal X variables
(multiple partial correlations)

Variable	dbRDA1	dbRDA2	dbRDA3	dbRDA4	dbRDA5	dbRDA6	dbRDA7	dbRDA8
Pebble	0.228	-0.028	0.436	-0.664	0.076	0.412	0.171	-0.333
Granule	0.224	-0.315	-0.179	-0.111	0.775	-0.276	-0.321	-0.161
Very Coarse Sand	-0.145	0.623	-0.124	-0.434	-0.069	-0.119	-0.601	0.08
Medium Sand	-0.034	-0.007	0.06	-0.443	0.037	-0.602	0.483	0.449
Fine Sand	0.505	0.169	-0.547	-0.076	0.102	0.436	0.179	0.423
Very Fine Sand	0.27	0.229	-0.42	-0.02	-0.23	-0.338	0.278	-0.673
Fine Sand	0.525	-0.442	0.076	-0.107	-0.54	-0.198	-0.405	0.132
Salinity	0.521	0.485	0.527	0.38	0.18	-0.183	0.018	0.072

Weights
(Coefficients for linear combinations of X's in the formation of dbRDA coordinates)

Variable	dbRDA1	dbRDA2	dbRDA3	dbRDA4	dbRDA5	dbRDA6	dbRDA7	dbRDA8
Pebble	-0.47618	10.101	6.6911	-8.8928	-5.1978	7.3163	1.7243	-1.9681
Granule	9.1574	-38.071	-7.2985	7.3852	17.701	-9.3983	0.055445	-1.8219
Very Coarse Sand	-2.9099	35.714	0.22487	-7.458	-10.163	4.5309	-4.938	2.6515
Medium Sand	2.4927	-9.4741	1.1431	-4.0982	0.99962	-10.199	3.7088	2.049
Fine Sand	5.9137	11.09	-4.2864	-2.327	-1.2429	7.4945	-2.0304	3.8148
Very Fine Sand	2.6127	3.257	-6.1814	0.10193	-1.044	-7.551	4.599	-7.1373
Fine Sand	4.0504	-8.3735	-0.074092	-3.0247	-4.1697	-1.8861	-2.4291	2.2105
Salinity	12.408	19.263	8.8855	6.0368	2.8236	-2.4541	0.24526	0.78189

d) Subaé

Specified solution

R2	RSS	N. Vars
		7
0.24885	134810.00	1;3;4;6-9

Marginal tests

Variable	SS (trace)	Pseudo-F	P	Prop.
Pebble	7254.5	2.6959	0.001	0.040421
Very Coarse Sand	4776.2	1.7498	0.048	0.026612
Coarse Sand	10573	4.0064	0.001	0.058912
Fine Sand	4832.4	1.7709	0.047	0.026925
Very Fine Sand	10988	4.1737	0.001	0.061221
Fine Sand	10040	3.7922	0.001	0.055939
Salinity	12629	4.8442	0.001	0.070365

% of var. expl. by ind. axes

Axis	% out of fitted model Ind.	Cum.	% out of total variation Ind.	Cum.
1	40.25	40.25	10.02	10.02
2	24.44	64.69	6.08	16.1
3	13.82	78.52	3.44	19.54
4	8.16	86.68	2.03	21.57
5	6.32	93	1.57	23.14
6	4.97	97.97	1.24	24.38
7	2.03	100	0.51	24.88

Sequential tests SEQUENTIAL TESTS

Variable	R^2	SS (trace)	Pseudo-F	P	Prop.	Cumul.	res.df
Pebble	0.040421	7254.5	2.6959	0.004	0.040421	0.040421	64
Very Coarse Sand	0.08001	7105.4	2.7111	0.006	0.03959	0.08001	63
Coarse Sand	0.11437	6167.5	2.4057	0.01	0.034364	0.11437	62
Fine Sand	0.14628	5726.7	2.2799	0.012	0.031908	0.14628	61
Very Fine Sand	0.17155	4534.8	1.83	0.028	0.025267	0.17155	60
Fine Sand	0.1978	4710.6	1.9303	0.033	0.026246	0.1978	59
Salinity	0.24885	9162.2	3.9418	0.001	0.05105	0.24885	58

Relationships between dbRDA coordinate axes and orthonormal X variables
(multiple partial correlations)

Variable	dbRDA1	dbRDA2	dbRDA3	dbRDA4	dbRDA5	dbRDA6	dbRDA7
Pebble	0.386	-0.017	-0.814	-0.304	0.088	0.013	0.298
Very Coarse Sand	0.09	0.155	0.031	-0.427	0.55	0.289	-0.632
Coarse Sand	-0.498	0.263	0.001	-0.264	-0.099	0.67	0.393
Fine Sand	0.239	0.073	0.488	-0.328	0.448	-0.252	0.573
Very Fine Sand	0.402	-0.319	0.285	-0.499	-0.598	0.197	-0.101
Fine Sand	0.076	-0.756	0.033	0.297	0.346	0.444	0.131
Salinity	0.609	0.478	0.128	0.46	-0.041	0.41	0.051

Weights
(Coefficients for linear combinations of X's in the formation of dbRDA coordinates)

Variable	dbRDA1	dbRDA2	dbRDA3	dbRDA4	dbRDA5	dbRDA6	dbRDA7
Pebble	5.9169	-1.0374	-11.188	-2.2284	-1.0787	-0.29135	3.3313
Very Coarse Sand	5.24	0.713	5.9308	-3.2833	6.8408	0.19502	-5.1015
Coarse Sand	-8.491	0.70006	-1.0497	-0.71331	-2.9572	5.7156	4.0123
Fine Sand	3.0978	0.26158	3.9209	-1.9337	5.5169	-0.86101	2.1612
Very Fine Sand	3.186	-2.6885	1.6215	-4.9933	-5.8231	1.2414	-1.0287
Fine Sand	-2.6589	-6.4063	0.63414	1.4071	3.8247	2.8472	1.3239
Salinity	12.895	11.079	2.0178	5.7377	-0.058688	3.9051	0.43538

Table 3S. Station-level temporal dissimilarity and main taxonomic contributors between consecutive surveys based on SIMPER analyses. **Mean BC** = mean Bray–Curtis dissimilarity between the two surveys; **First contributor** = taxon contributing most to that temporal dissimilarity; **Second contributor** = taxon with the second most contribution to that temporal dissimilarity; **Third contributor** = taxon with the third most contribution to that temporal dissimilarity; I**increase / decline / appearance / disappearance / no change** describes the direction of change. For example, Jaguaripe Station 1 from 2006 to 2007 had **BC dissimilarity = 0.761**, primarily associated with declines in Anthuridae (14.7%) and Amphipoda (12.3%) and an increase in Cirratulidae (8.6%).

Estuary	Station	Earlier	Later	Mean BC	First contributor	Second contributor	Third contributor
Jaguaripe	1	2006-05	2007-08	0.761	Anthuridae (14.7%, decline)	Amphipoda (12.3%, decline)	Cirratulidae (8.6%, increase)
Jaguaripe	1	2007-08	2010-07	0.719	Cirratulidae (8.9%, decline)	Branchiostomatidae (7.2%, increase)	Spionidae (7.1%, appearance)
Jaguaripe	1	2010-07	2014-08	0.684	Cirratulidae (7.8%, decline)	Branchiostomatidae (7.4%, increase)	Tanaidacea (6.7%, increase)
Jaguaripe	1	2014-08	2019-03	0.756	Branchiostomatidae (9.7%, disappearance)	Cirratulidae (9.2%, increase)	Paraonidae (9.2%, increase)
Jaguaripe	1	2019-03	2022-07	0.750	Paraonidae (8.2%, decline)	Lumbrineridae (6.1%, appearance)	Nemertea (6.1%, appearance)
Jaguaripe	2	2006-05	2007-08	0.708	Glyceridae (14.7%, increase)	Orbiniidae (12.9%, increase)	Cirratulidae (12.7%, increase)
Jaguaripe	2	2007-08	2010-07	0.861	Orbiniidae (9.3%, decline)	Maldanidae (8.7%, appearance)	Paraonidae (7.9%, appearance)
Jaguaripe	2	2010-07	2014-08	0.681	Maldanidae (7.7%, decline)	Magelonidae (7.6%, disappearance)	Branchiostomatidae (6.6%, increase)
Jaguaripe	2	2014-08	2019-03	0.840	Cirratulidae (10.8%, disappearance)	Branchiostomatidae (7.0%, increase)	Amphipoda (6.1%, disappearance)
Jaguaripe	2	2019-03	2022-07	0.829	Caecidae (8.7%, increase)	Orbiniidae (6.6%, increase)	Paraonidae (6.2%, increase)
Jaguaripe	3	2006-05	2007-08	0.478	Orbiniidae (17.1%, increase)	Nereididae (10.4%, decline)	Poecilochaetidae (10.2%, disappearance)
Jaguaripe	3	2007-08	2010-07	0.954	Magelonidae (17.9%, decline)	Orbiniidae (17.6%, disappearance)	Cirratulidae (15.5%, disappearance)
Jaguaripe	3	2010-07	2014-08	0.916	Cirratulidae (11.1%, appearance)	Magelonidae (10.2%, increase)	Nemertea (7.2%, appearance)
Jaguaripe	3	2014-08	2019-03	0.561	Nemertea (7.6%, decline)	Trichobranchidae (7.3%, disappearance)	Cirratulidae (5.2%, decline)
Jaguaripe	3	2019-03	2022-07	0.661	Magelonidae (9.3%, increase)	Pilargidae (5.6%, increase)	Orbiniidae (5.4%, increase)
Jaguaripe	4	2006-05	2007-08	0.374	Orbiniidae (21.8%, increase)	Goniadidae (10.8%, disappearance)	Magelonidae (10.6%, increase)
Jaguaripe	4	2007-08	2010-07	0.965	Magelonidae (33.1%, decline)	Orbiniidae (29.1%, disappearance)	Paraonidae (7.0%, appearance)
Jaguaripe	4	2010-07	2014-08	0.828	Paraonidae (28.5%, increase)	Goniadidae (13.3%, appearance)	Pilargidae (12.5%, appearance)
Jaguaripe	4	2014-08	2019-03	0.568	Capitellidae (16.6%, increase)	Paraonidae (13.7%, increase)	Magelonidae (13.2%, increase)
Jaguaripe	4	2019-03	2022-07	0.721	Paraonidae (18.4%, disappearance)	Orbiniidae (15.8%, increase)	Nemertea (9.5%, increase)
Jaguaripe	5	2006-05	2007-08	0.440	Tellinidae (22.9%, decline)	Magelonidae (22.0%, decline)	Orbiniidae (20.3%, no change)
Jaguaripe	5	2007-08	2010-07	0.876	Magelonidae (30.1%, decline)	Tellinidae (13.5%, decline)	Glyceridae (10.1%, disappearance)
Jaguaripe	5	2010-07	2014-08	0.848	Tellinidae (24.0%, increase)	Glyceridae (16.3%, appearance)	Paraonidae (13.0%, decline)
Jaguaripe	5	2014-08	2019-03	0.768	Paraonidae (23.0%, increase)	Magelonidae (22.7%, increase)	Capitellidae (14.5%, increase)
Jaguaripe	5	2019-03	2022-07	0.706	Paraonidae (16.5%, disappearance)	Magelonidae (16.2%, disappearance)	Spionidae (12.0%, increase)
Jaguaripe	6	2006-05	2007-08	0.665	Tellinidae (44.0%, decline)	Glyceridae (19.3%, increase)	Pilargidae (16.9%, appearance)
Jaguaripe	6	2007-08	2010-07	0.900	Tellinidae (23.5%, decline)	Paraonidae (16.4%, appearance)	Glyceridae (11.7%, disappearance)
Jaguaripe	6	2010-07	2014-08	0.891	Spionidae (16.0%, appearance)	Tellinidae (13.5%, increase)	Capitellidae (10.1%, increase)
Jaguaripe	6	2014-08	2019-03	0.711	Magelonidae (14.6%, appearance)	Solecurtidae (13.5%, appearance)	Capitellidae (12.2%, increase)
Jaguaripe	6	2019-03	2022-07	0.670	Spionidae (15.2%, increase)	Solecurtidae (13.6%, disappearance)	Magelonidae (13.4%, decline)
Jaguaripe	7	2006-05	2007-08	0.724	Tellinidae (47.7%, decline)	Nereididae (18.2%, appearance)	Orbiniidae (10.1%, disappearance)
Jaguaripe	7	2007-08	2010-07	0.837	Tellinidae (23.7%, increase)	Capitellidae (20.2%, appearance)	Nereididae (19.6%, disappearance)
Jaguaripe	7	2010-07	2014-08	0.797	Nereididae (27.3%, appearance)	Nemertea (16.2%, appearance)	Tellinidae (14.9%, increase)
Jaguaripe	7	2014-08	2019-03	0.843	Nereididae (26.4%, disappearance)	Nemertea (15.7%, disappearance)	Tellinidae (14.3%, disappearance)
Jaguaripe	7	2019-03	2022-07	0.872	Chironomidae(larva) (19.6%, appearance)	Spionidae (11.6%, increase)	Tellinidae (11.1%, appearance)
Jaguaripe	8	2006-05	2007-08	0.736	Nereididae (53.5%, increase)	Tellinidae (20.0%, disappearance)	Veneridae (19.9%, disappearance)
Jaguaripe	8	2007-08	2010-07	0.465	Nereididae (53.8%, increase)	Capitellidae (19.8%, appearance)	Solecurtidae (13.1%, appearance)
Jaguaripe	8	2010-07	2014-08	0.710	Nereididae (23.4%, decline)	Spionidae (18.4%, appearance)	Capitellidae (15.5%, increase)
Jaguaripe	8	2014-08	2019-03	0.718	Capitellidae (14.2%, decline)	Nemertea (13.6%, disappearance)	Nereididae (13.2%, disappearance)
Jaguaripe	8	2019-03	2022-07	0.704	Tellinidae (13.7%, increase)	Nereididae (12.2%, appearance)	Capitellidae (10.9%, increase)
Jaguaripe	9	2006-05	2007-08	0.254	Nereididae (85.7%, increase)	Gammaridea (14.3%, appearance)	Cirratulidae (0.0%, no change)
Jaguaripe	9	2007-08	2010-07	0.828	Nereididae (63.6%, decline)	Chironomidae(larva) (15.2%, appearance)	Poecilochaetidae (6.9%, appearance)
Jaguaripe	9	2010-07	2014-08	0.870	Nereididae (28.6%, increase)	Nemertea (17.1%, appearance)	Oligochaeta (13.5%, appearance)
Jaguaripe	9	2014-08	2019-03	1.000	Nereididae (35.9%, disappearance)	Nemertea (18.0%, disappearance)	Oligochaeta (13.8%, disappearance)
Jaguaripe	9	2019-03	2022-07	1.000	Nereididae (44.8%, appearance)	Chironomidae(larva) (23.0%, appearance)	Rissoidae (19.4%, appearance)
Jaguaripe	10	2006-05	2007-08	1.000	Nereididae (100.0%, appearance)	Oenonidae (0.0%, no change)	Cirratulidae (0.0%, no change)
Jaguaripe	10	2007-08	2010-07	0.263	Nereididae (85.1%, increase)	Chironomidae(larva) (14.9%, appearance)	Cirratulidae (0.0%, no change)
Jaguaripe	10	2010-07	2014-08	0.392	Nereididae (35.1%, increase)	Chironomidae(larva) (17.5%, increase)	Ostracoda (11.3%, appearance)
Jaguaripe	10	2014-08	2019-03	1.000	Nereididae (63.9%, disappearance)	Chironomidae(larva) (8.1%, disappearance)	Semelidae (6.0%, disappearance)
Jaguaripe	10	2019-03	2022-07	1.000	Nereididae (50.7%, appearance)	Chironomidae(larva) (24.7%, appearance)	Rissoidae (14.5%, appearance)
Paraguaçu	1	2005-05	2005-12	0.556	Nuculidae (11.3%, decline)	Amphiuridae (10.7%, decline)	Corbulidae (9.5%, appearance)
Paraguaçu	1	2005-12	2011-06	0.801	Cirratulidae (11.3%, decline)	Spionidae (10.2%, appearance)	Nuculidae (8.4%, decline)
Paraguaçu	1	2011-06	2014-08	1.000	Ophiuroidea (20.5%, disappearance)	Spionidae (16.6%, disappearance)	Nuculidae (13.9%, disappearance)
Paraguaçu	1	2014-08	2022-10	1.000	Cirratulidae (17.0%, appearance)	Sipuncula (7.7%, appearance)	Ophiactidae (5.8%, appearance)
Paraguaçu	2	2005-05	2005-12	0.848	Amphiuridae (13.5%, increase)	Tellinidae (10.2%, increase)	Glyceridae (7.9%, increase)
Paraguaçu	2	2005-12	2011-06	0.807	Spionidae (6.6%, appearance)	Poecilochaetidae (6.4%, appearance)	Nereididae (5.8%, increase)
Paraguaçu	2	2011-06	2014-08	0.507	Sipuncula (7.1%, increase)	Gammaridea (5.4%, appearance)	Onuphidae (4.8%, decline)
Paraguaçu	2	2014-08	2022-10	0.742	Poecilochaetidae (5.2%, disappearance)	Magelonidae (4.6%, disappearance)	Sipuncula (4.4%, decline)
Paraguaçu	3	2005-05	2005-12	0.836	Bryozoa (23.1%, decline)	Nuculidae (23.0%, increase)	Tellinidae (12.9%, increase)
Paraguaçu	3	2005-12	2011-06	0.770	Nuculidae (14.4%, disappearance)	Poecilochaetidae (13.6%, increase)	Tellinidae (10.3%, increase)
Paraguaçu	3	2011-06	2014-08	0.931	Goneplacidae (12.5%, appearance)	Corbulidae (11.7%, appearance)	Cirratulidae (5.6%, decline)
Paraguaçu	3	2014-08	2022-10	0.949	Corbulidae (10.7%, disappearance)	Goneplacidae (9.8%, disappearance)	Nuculidae (9.7%, appearance)
Paraguaçu	4	2005-05	2005-12	0.906	Nuculidae (13.0%, disappearance)	Paguridae (12.9%, decline)	Tellinidae (12.4%, no change)
Paraguaçu	4	2005-12	2011-06	0.956	Orbiniidae (13.7%, appearance)	Corbulidae (9.9%, disappearance)	Cirratulidae (9.6%, increase)

Paraguaçu	4	2011-06	2014-08	0.911	Trochochaetidae (11.4%, appearance)	Cirratulidae (9.5%, increase)	Gammaridea (9.1%, appearance)
Paraguaçu	4	2014-08	2022-10	0.867	Trochochaetidae (8.1%, decline)	Cirratulidae (7.4%, decline)	Gammaridea (6.7%, disappearance)
Paraguaçu	5	2005-05	2005-12	0.933	Cirratulidae (17.6%, disappearance)	Bryozoa (14.0%, decline)	Tellinidae (9.6%, decline)
Paraguaçu	5	2005-12	2011-06	0.935	Nereididae (7.1%, appearance)	Poecilochaetidae (6.8%, appearance)	Spionidae (6.7%, appearance)
Paraguaçu	5	2011-06	2014-08	0.589	Nemertea (6.0%, increase)	Tellinidae (4.7%, decline)	Orbiniidae (4.5%, decline)
Paraguaçu	5	2014-08	2022-10	0.849	Poecilochaetidae (8.2%, disappearance)	Nereididae (6.3%, disappearance)	Cirratulidae (6.3%, decline)
Paraguaçu	6	2005-05	2005-12	0.822	Glyceridae (9.8%, appearance)	Malletiidae (8.1%, appearance)	Tellinidae (6.5%, increase)
Paraguaçu	6	2005-12	2011-06	0.773	Spionidae (7.7%, increase)	Sternaspidae (6.3%, appearance)	Paraonidae (5.9%, appearance)
Paraguaçu	6	2011-06	2014-08	0.551	Spionidae (4.6%, decline)	Solecurtidae (4.2%, appearance)	Orbiniidae (4.1%, disappearance)
Paraguaçu	6	2014-08	2022-10	0.610	Sternaspidae (4.9%, decline)	Syllidae (4.0%, appearance)	Corbulidae (3.9%, increase)
Paraguaçu	7	2005-05	2005-12	0.783	Tellinidae (27.9%, decline)	Glyceridae (19.1%, decline)	Hesionidae (14.1%, no change)
Paraguaçu	7	2005-12	2011-06	0.929	Paraonidae (15.7%, appearance)	Pilargidae (10.3%, appearance)	Magelonidae (9.1%, appearance)
Paraguaçu	7	2011-06	2014-08	0.620	Pilargidae (8.2%, increase)	Magelonidae (6.8%, decline)	Goneplacidae (6.6%, appearance)
Paraguaçu	7	2014-08	2022-10	0.747	Pilargidae (12.0%, decline)	Spionidae (6.1%, increase)	Paraonidae (5.7%, decline)
Paraguaçu	8	2005-05	2005-12	0.792	Veneridae (32.5%, appearance)	Tellinidae (16.6%, decline)	Donacidae (10.4%, decline)
Paraguaçu	8	2005-12	2011-06	0.763	Veneridae (31.7%, disappearance)	Tellinidae (13.2%, increase)	Nemertea (10.7%, appearance)
Paraguaçu	8	2011-06	2014-08	0.780	Glyceridae (11.9%, increase)	Nereididae (11.2%, appearance)	Oligochaeta (9.5%, appearance)
Paraguaçu	8	2014-08	2022-10	0.720	Nemertea (20.0%, appearance)	Nereididae (11.7%, disappearance)	Oligochaeta (11.5%, decline)
Paraguaçu	9	2005-05	2005-12	0.891	Nereididae (28.7%, disappearance)	Veneridae (23.3%, appearance)	Tellinidae (18.8%, decline)
Paraguaçu	9	2005-12	2011-06	0.857	Veneridae (23.6%, decline)	Tellinidae (16.0%, increase)	Spionidae (9.1%, appearance)
Paraguaçu	9	2011-06	2014-08	0.874	Oligochaeta (13.7%, appearance)	Glyceridae (8.6%, increase)	Paraonidae (7.8%, appearance)
Paraguaçu	9	2014-08	2022-10	0.788	Nemertea (14.1%, appearance)	Oligochaeta (11.1%, decline)	Glyceridae (7.4%, decline)
Paraguaçu	10	2005-05	2005-12	0.544	Veneridae (32.2%, appearance)	Tellinidae (23.9%, decline)	Cirolanidae (17.4%, decline)
Paraguaçu	10	2005-12	2011-06	0.707	Veneridae (28.6%, disappearance)	Cirolanidae (25.1%, increase)	Tellinidae (14.2%, decline)
Paraguaçu	10	2011-06	2014-08	0.742	Cirolanidae (20.0%, decline)	Glyceridae (16.1%, appearance)	Tellinidae (11.5%, increase)
Paraguaçu	10	2014-08	2022-10	0.541	Ostracoda (18.7%, appearance)	Cirolanidae (10.3%, increase)	Nemertea (9.4%, decline)
Subaé	1	2004-06	2006-03	0.973	Sternaspidae (42.5%, disappearance)	Gammaridea (9.5%, disappearance)	Spionidae (9.2%, no change)
Subaé	1	2006-03	2009-12	0.940	Orbiniidae (15.5%, decline)	Tellinidae (14.2%, appearance)	Spionidae (12.5%, disappearance)
Subaé	1	2009-12	2011-04	0.846	Tellinidae (22.0%, decline)	Orbiniidae (18.5%, increase)	Glyceridae (18.1%, decline)
Subaé	1	2011-04	2013-03	0.901	Cirratulidae (18.2%, appearance)	Paraonidae (17.2%, appearance)	Lucinidae (11.6%, appearance)
Subaé	1	2013-03	2022-12	0.961	Nemertea (15.6%, appearance)	Cirratulidae (7.5%, increase)	Magelonidae (6.6%, appearance)
Subaé	2	2004-06	2006-03	0.702	Ostreoidea (12.4%, disappearance)	Porifera (6.2%, appearance)	Maldanidae (5.2%, decline)
Subaé	2	2006-03	2009-12	0.808	Sabellidae (6.3%, appearance)	Cirratulidae (6.2%, increase)	Syllidae (5.6%, increase)
Subaé	2	2009-12	2011-04	0.953	Cirratulidae (8.0%, disappearance)	Syllidae (6.6%, disappearance)	Sabellidae (6.5%, disappearance)
Subaé	2	2011-04	2013-03	0.719	Orbiniidae (26.7%, disappearance)	Polynoidae (18.6%, disappearance)	Goniadidae (11.3%, disappearance)
Subaé	2	2013-03	2022-12	0.998	Aoridae (6.9%, appearance)	Syllidae (6.8%, appearance)	Sternaspidae (5.3%, appearance)
Subaé	3	2004-06	2006-03	0.846	Sternaspidae (39.9%, decline)	Glyceridae (16.4%, appearance)	Capitellidae (16.0%, disappearance)
Subaé	3	2006-03	2009-12	0.938	Penaeoidea (37.9%, appearance)	Glyceridae (14.3%, disappearance)	Capitellidae (12.2%, appearance)
Subaé	3	2009-12	2011-04	0.979	Penaeoidea (26.7%, disappearance)	Corbulidae (13.0%, appearance)	Goniadidae (10.1%, appearance)
Subaé	3	2011-04	2013-03	0.888	Goniadidae (19.6%, decline)	Corbulidae (18.9%, disappearance)	Nereididae (14.4%, no change)
Subaé	3	2013-03	2022-12	0.740	Goniadidae (22.9%, no change)	Nereididae (18.7%, no change)	Branchiostomatidae (13.4%, appearance)
Subaé	4	2004-06	2006-03	0.904	Capitellidae (15.1%, disappearance)	Sternaspidae (14.4%, disappearance)	Goniadidae (13.8%, decline)
Subaé	4	2006-03	2009-12	0.786	Arcidae (20.5%, appearance)	Syllidae (17.1%, appearance)	Alpheidae (17.1%, appearance)
Subaé	4	2009-12	2011-04	0.984	Goniadidae (25.0%, appearance)	Psammobiidae (14.5%, appearance)	Magelonidae (8.3%, appearance)
Subaé	4	2011-04	2013-03	0.984	Goniadidae (23.7%, disappearance)	Psammobiidae (13.6%, disappearance)	Magelonidae (8.1%, disappearance)
Subaé	4	2013-03	2022-12	0.919	Sipuncula (13.3%, increase)	Vitrinellidae (12.4%, appearance)	Mytilidae (9.5%, appearance)
Subaé	5	2004-06	2006-03	0.250	Spionidae (50.0%, appearance)	Porifera (50.0%, appearance)	Cirratulidae (0.0%, no change)
Subaé	5	2006-03	2009-12	0.750	Magelonidae (19.4%, appearance)	Orbiniidae (17.2%, appearance)	Goniadidae (16.5%, appearance)
Subaé	5	2009-12	2011-04	0.804	Orbiniidae (19.4%, decline)	Goniadidae (18.4%, decline)	Magelonidae (13.0%, disappearance)
Subaé	5	2011-04	2013-03	0.803	Goniadidae (25.8%, increase)	Onuphidae (13.8%, no change)	Orbiniidae (11.0%, disappearance)
Subaé	5	2013-03	2022-12	0.980	Orbiniidae (15.0%, appearance)	Sipuncula (14.3%, appearance)	Nemertea (13.5%, appearance)
Subaé	6	2004-06	2006-03	0.873	Capitellidae (26.8%, disappearance)	Orbiniidae (24.5%, decline)	Goniadidae (12.1%, increase)
Subaé	6	2006-03	2009-12	0.844	Magelonidae (25.2%, increase)	Goniadidae (19.3%, disappearance)	Glyceridae (16.3%, appearance)
Subaé	6	2009-12	2011-04	0.861	Magelonidae (32.6%, disappearance)	Glyceridae (23.8%, decline)	Orbiniidae (16.2%, decline)
Subaé	6	2011-04	2013-03	0.913	Goniadidae (20.5%, increase)	Orbiniidae (11.0%, disappearance)	Nereididae (9.9%, appearance)
Subaé	6	2013-03	2022-12	0.920	Pilargidae (13.1%, appearance)	Capitellidae (12.4%, increase)	Goniadidae (11.7%, decline)
Subaé	7	2004-06	2006-03	0.795	Tellinidae (30.3%, increase)	Nemertea (15.6%, disappearance)	Goniadidae (14.2%, disappearance)
Subaé	7	2006-03	2009-12	0.926	Tellinidae (14.1%, disappearance)	Sternaspidae (10.2%, appearance)	Solecurtidae (10.1%, appearance)
Subaé	7	2009-12	2011-04	0.960	Tellinidae (28.7%, appearance)	Sternaspidae (9.6%, disappearance)	Solecurtidae (9.4%, disappearance)
Subaé	7	2011-04	2013-03	0.582	Tellinidae (19.5%, increase)	Orbiniidae (18.6%, increase)	Pilargidae (15.1%, increase)
Subaé	7	2013-03	2022-12	0.775	Tellinidae (17.8%, decline)	Solecurtidae (11.6%, appearance)	Orbiniidae (9.1%, decline)
Subaé	8	2004-06	2006-03	0.658	Tellinidae (79.2%, decline)	Orbiniidae (20.8%, appearance)	Cirratulidae (0.0%, no change)
Subaé	8	2006-03	2009-12	0.808	Tellinidae (75.2%, increase)	Pilargidae (9.1%, appearance)	Orbiniidae (6.7%, disappearance)
Subaé	8	2009-12	2011-04	0.385	Tellinidae (60.4%, decline)	Pilargidae (15.0%, disappearance)	Syllidae (7.4%, disappearance)
Subaé	8	2011-04	2013-03	0.479	Tellinidae (44.5%, decline)	Pilargidae (21.3%, appearance)	Mytilidae (13.5%, increase)
Subaé	8	2013-03	2022-12	0.612	Nemertea (36.7%, appearance)	Tellinidae (17.8%, decline)	Pilargidae (15.9%, disappearance)
Subaé	9	2004-06	2006-03	0.812	Nereididae (60.2%, disappearance)	Tellinidae (30.2%, appearance)	Mytilidae (9.6%, appearance)
Subaé	9	2006-03	2009-12	0.799	Tellinidae (91.8%, increase)	Capitellidae (4.6%, appearance)	Mytilidae (3.6%, disappearance)
Subaé	9	2009-12	2011-04	1.000	Tellinidae (93.1%, disappearance)	Capitellidae (4.1%, disappearance)	Chironomidae(larva) (2.9%, appearance)
Subaé	9	2011-04	2013-03	0.891	Capitellidae (34.9%, appearance)	Tellinidae (32.8%, appearance)	Pilargidae (19.0%, appearance)
Subaé	9	2013-03	2022-12	0.930	Nemertea (26.9%, appearance)	Capitellidae (19.0%, disappearance)	Semelidae (18.7%, appearance)
Subaé	10	2004-06	2006-03	0.756	Pilargidae (55.3%, appearance)	Nereididae (29.2%, increase)	Spionidae (15.5%, appearance)
Subaé	10	2006-03	2009-12	0.970	Tellinidae (59.4%, appearance)	Pilargidae (16.7%, disappearance)	Nereididae (10.1%, decline)
Subaé	10	2009-12	2011-04	0.931	Tellinidae (77.5%, decline)	Nereididae (9.2%, decline)	Spionidae (7.0%, disappearance)
Subaé	10	2011-04	2013-03	0.773	Capitellidae (28.0%, appearance)	Ampharetidae (21.6%, appearance)	Spionidae (18.9%, appearance)
Subaé	10	2013-03	2022-12	0.834	Capitellidae (24.8%, disappearance)	Ampharetidae (18.2%, disappearance)	Spionidae (16.7%, disappearance)
Subaé	11	2004-06	2006-03	1.000	Nereididae (56.2%, appearance)	Psammobiidae (21.9%, appearance)	Capitellidae (16.2%, appearance)
Subaé	11	2006-03	2009-12	0.716	Capitellidae (32.2%, increase)	Tellinidae (17.9%, appearance)	Spionidae (14.6%, appearance)
Subaé	11	2009-12	2011-04	0.949	Capitellidae (39.3%, decline)	Nereididae (20.1%, disappearance)	Tellinidae (17.5%, disappearance)
Subaé	11	2011-04	2013-03	0.756	Nereididae (37.7%, appearance)	Capitellidae (29.1%, increase)	Oligochaeta (15.5%, appearance)
Subaé	11	2013-03	2022-12	0.767	Nereididae (27.4%, increase)	Ampharetidae (27.1%, appearance)	Oligochaeta (15.5%, increase)

Table 4S. Most recurrent taxa contributing to station-level temporal dissimilarity between consecutive surveys based on SIMPER analyses (Summary of Table 3S).

Estuary	Earlier	Later	Most recurrent contributors	Interpretation
Jaguaripe	2006-05	2007-08	Nereididae (5 stations; appearance 2, increase 2, decline 1); Orbiniidae (5 stations; increase 3, no change 1, disappearance 1); Tellinidae (4 stations; decline 3, disappearance 1)	Different directional counts indicate station-level redistribution.
Jaguaripe	2007-08	2010-07	Nereididae (4 stations; increase 2, disappearance 1, decline 1); Magelonidae (3 stations; decline 3); Tellinidae (3 stations; decline 2, increase 1)	Different directional counts indicate station-level redistribution.
Jaguaripe	2010-07	2014-08	Nereididae (4 stations; increase 2, appearance 1, decline 1); Tellinidae (3 stations; increase 3); Nemertea (3 stations; appearance 3)	Different directional counts indicate station-level redistribution.
Jaguaripe	2014-08	2019-03	Nereididae (4 stations; disappearance 4); Capitellidae (4 stations; increase 3, decline 1); Nemertea (4 stations; disappearance 3, decline 1)	Different directional counts indicate station-level redistribution.
Jaguaripe	2019-03	2022-07	Paraonidae (4 stations; disappearance 2, decline 1, increase 1); Nereididae (3 stations; appearance 3); Chironomidae(larva) (3 stations; appearance 3)	Different directional counts indicate station-level redistribution.
Paraguaçu	2005-05	2005-12	Tellinidae (9 stations; decline 5, increase 3, no change 1); Veneridae (3 stations; appearance 3); Nuculidae (3 stations; decline 1, increase 1, disappearance 1)	Different directional counts indicate station-level redistribution.
Paraguaçu	2005-12	2011-06	Spionidae (5 stations; appearance 4, increase 1); Tellinidae (4 stations; increase 3, decline 1); Veneridae (3 stations; disappearance 2, decline 1)	Different directional counts indicate station-level redistribution.
Paraguaçu	2011-06	2014-08	Glyceridae (3 stations; increase 2, appearance 1); Oligochaeta (2 stations; appearance 2); Spionidae (2 stations; disappearance 1, decline 1)	Different directional counts indicate station-level redistribution.
Paraguaçu	2014-08	2022-10	Nemertea (3 stations; appearance 2, decline 1); Cirratulidae (3 stations; decline 2, appearance 1); Oligochaeta (2 stations; decline 2)	Different directional counts indicate station-level redistribution.
Subaé	2004-06	2006-03	Capitellidae (4 stations; disappearance 3); Nereididae (3 stations; disappearance 1, increase 1, appearance 1); Tellinidae (3 stations; increase 1, decline 1, appearance 1)	Different directional counts indicate station-level redistribution.
Subaé	2006-03	2009-12	Tellinidae (6 stations; appearance 3, increase 2, disappearance 1); Capitellidae (3 stations; appearance 1, increase 1); Orbiniidae (3 stations; decline 1, appearance 1)	Different directional counts indicate station-level redistribution.
Subaé	2009-12	2011-04	Tellinidae (6 stations; decline 3, disappearance 2, appearance 1); Orbiniidae (3 stations; decline 2, increase 1); Magelonidae (3 stations; disappearance 2, appearance 1)	Different directional counts indicate station-level redistribution.
Subaé	2011-04	2013-03	Goniadidae (5 stations; disappearance 2, increase 2, decline 1); Orbiniidae (4 stations; disappearance 3, increase 1); Tellinidae (3 stations; increase 1, decline 1, appearance 1)	Different directional counts indicate station-level redistribution.
Subaé	2013-03	2022-12	Nemertea (4 stations; appearance 4); Capitellidae (3 stations; disappearance 2, increase 1); Nereididae (2 stations; no change 1, increase 1)	Different directional counts indicate station-level redistribution.
